# PolyQ-expanded ataxin-3 is associated with mitochondrial alterations in Machado–Joseph disease

**DOI:** 10.1242/dmm.052757

**Published:** 2026-09-18

**Authors:** Ignacio Simó, Katherine J. Robinson, Julia Y. Kam, Yvonne Wang, Andrea Kuriakose, Esmeralda Paric, Flora Cheng, Anastasiya Potapenko, Yousun An, Maxinne Watchon, Albert Lee, Angela S. Laird

**Affiliations:** Motor Neuron Disease Research Centre, Macquarie Medical School, Faculty of Medicine, Health and Human Sciences, Macquarie University, Sydney, NSW 2109, Australia

**Keywords:** Mitochondria, Neurodegeneration, Polyglutamine, Trinucleotide repeat disorder

## Abstract

Spinocerebellar ataxia type 3, also known as Machado–Joseph disease (MJD), is a fatal neurodegenerative disease caused by an expanded CAG repeat in ataxin-3 (*ATXN3*). Here, we investigated mitochondrial alterations across complementary MJD models, including transgenic zebrafish, CMVMJD135 mice and primary neuronal cultures. Proteomic profiling of brain lysates from male and female wild-type and CMVMJD135 mice identified mitochondrial alterations and altered oxidative phosphorylation-associated protein abundance as major shared features in both male and female MJD mice. Primary neuron cultures derived from CMVMJD135 mice were examined, validating the proteomic findings and revealing alterations in mitochondrial morphology. We further examined a transgenic zebrafish model of MJD that expresses EGFP-fused human ataxin-3 containing 84 glutamines in neurons (driven by the pan-neuronal elavl3/HuC promoter). The MJD zebrafish model also exhibited altered levels of mitochondrial electron transport chain complex proteins and enhanced sensitivity to rotenone. Notably, complex II-associated alterations featured across most assays, supporting complex II-linked dysregulation as a convergent, potentially targetable component of MJD. These phenotypes provide a robust platform for evaluating potential mitochondrial-targeted therapies and support growing evidence that disrupted mitochondrial homeostasis contributes to MJD pathogenesis.

## Teaser

**Summary:** Complementary mouse and zebrafish models of Machado–Joseph disease show that polyQ-expanded ataxin-3 is associated with mitochondrial alterations, with complex II-linked metabolic disruption as a consistent feature.

## INTRODUCTION

Spinocerebellar ataxia type 3, also known as Machado–Joseph disease (MJD), is a progressive autosomal dominant neurodegenerative disorder caused by an expanded CAG trinucleotide repeat in the *ATXN3* gene. This results in a long polyglutamine (polyQ) tract within the ataxin-3 (ATXN3) protein, leading to protein misfolding, aggregation and cellular toxicity (Costa and Paulson, 2012). MJD is characterised by symptoms of ataxia (loss of balance and coordination), resulting in an unsteady gait, clumsiness and difficulties with fine motor tasks (Costa and Paulson, 2012). Despite extensive research, the exact mechanisms driving neurodegeneration in MJD still remain unclear. Emerging evidence points to mitochondrial dysfunction as a central contributor to neurodegenerative disease pathology, particularly in neurons, which are highly reliant on mitochondrial integrity for survival and function (Johri and Beal, 2012).

Mitochondria are critical for maintaining neuronal homeostasis, serving as the primary source of adenosine triphosphate (ATP) production through oxidative phosphorylation, and playing a role in the regulation of calcium dynamics and mediation of apoptosis. Dysfunction in any of these processes can severely affect neuronal health, given the high metabolic demands of neurons and their limited capacity for regeneration. Mitochondrial deficits have been found to play a role in the pathogenesis and progression of a range of different neurodegenerative diseases, including Alzheimer's disease (Benarroch, 2009; Cobley et al., 2018), Huntington's disease (HD) (Jurcau and Jurcau, 2023) and amyotrophic lateral sclerosis (Zhao et al., 2022). Variants in genes such as superoxide dismutase 1 (*SOD1*), *C9orf72* and *CHCHD10* have not only been identified as causes of familial amyotrophic lateral sclerosis, but have also been closely associated with mitochondrial alterations, suggesting that impaired mitochondrial homeostasis plays a central role in disease pathogenesis (Tafuri et al., 2015; Mehta et al., 2021; Project MinE ALS Sequencing Consortium, 2018). Blockage of the mitochondrial electron transport chain (ETC), through administration of rotenone, also induces symptoms of Parkinson's disease, including selective nigrostriatal dopaminergic degeneration and α-synuclein-positive cytoplasmic inclusions (Betarbet et al., 2000; Sherer et al., 2003). Moreover, striatal neurodegeneration linked to mitochondrial dysregulation has been demonstrated in genetic and toxin-induced animal and cellular models of HD and post-mortem human brain tissue from patients with HD (Kim et al., 2010; Kodsi and Swerdlow, 1997).

Recently, Harmuth et al. (2022) reported that fibroblasts cultured from patients with MJD exhibit abnormal mitochondria, resulting in decreased ATP (cellular energy) production. Hsu et al. (2017) have previously reported that expression of truncated C-terminal fragments of mutant ataxin-3 disrupts the mitochondrial ETC, leading to impaired oxidative phosphorylation and ATP production (Hsu et al., 2017). Proteomic analyses of brain tissues from MJD mouse models have previously revealed downregulation of several ETC complexes, including complexes I and IV, further implicating energy metabolism defects in the disease process (Wiatr et al., 2021). In the Ki150 MJD mouse model, mutant ataxin-3 showed weakened interactions with numerous mitochondrial proteins including NDUF subunits, COX subunits and VDAC3, with altered mitochondrial motility along MJD neurites (Piasecki et al., 2023). Functionally, both the cerebellum from the CMVMJD135 mice (which express the full-length human ATXN3c isoform containing an expanded polyQ tract of 135 glutamines under the control of the CMV promoter) and the PC6-3 cell line (a rat PC12 subline stably expressing doxycycline-inducible human ATXN3 with an expanded polyQ tract of 108 glutamines) exhibit decreased mitochondrial respiration, ATP production, membrane potential and cytochrome c levels, with an additional compensatory glycolytic shift observed in cells (Almeida et al., 2023). Computational analyses have further predicted that interactions between ataxin-3 and key mitophagy regulators including parkin (PRKN), beclin-1 (BECN1) and TBK1 are disrupted in MJD, suggesting impaired mitochondrial quality control as an additional disease mechanism (Vieira et al., 2025). Indeed, Kristensen et al. (2018) have demonstrated that ataxin-3 interacts with mitochondrial proteins and reported stronger interactions of polyQ-expanded ataxin-3 than wild-type (WT) ataxin-3 with SDHB, NDUFA4 (also known as COXFA4) and COA7.

Mitochondrial dysregulation in MJD is not limited to impaired energy production. Abnormalities in mitochondrial morphology, such as mitochondrial fission events, have also been reported, suggesting that disruption of mitochondrial dynamics is a common characteristic in mouse and cell models expressing a truncated and aggregation-prone form of mutant ataxin-3 (Hsu et al., 2017). These morphological defects are accompanied by reduced levels of key mitochondrial proteins such as the mitofusins MFN1 and MFN2 (Hsu et al., 2017). Furthermore, mitochondrial trafficking, a process critical for distributing mitochondria to regions of high metabolic demand in neurons, appears to be compromised in MJD. Impaired trafficking disrupts synaptic function and likely contributes to the progressive degeneration observed in MJD (Piasecki et al., 2023). These findings align with observations from other polyQ diseases, such as HD, in which similar disruptions in mitochondrial transport and dynamics have been reported (Li et al., 2010).

In this study, we investigated the mitochondrial alterations associated with MJD using a combination of *in vivo* and *in vitro* models, including MJD zebrafish, primary mouse neuronal cultures (both previously unstudied in relation to mitochondrial function) and brain tissue from the CMVMJD135 mice that have been reported to have compromised mitochondrial respiration (Almeida et al., 2023). Using proteomic analyses, mitochondrial staining, metabolite measurements and stress-challenge assays, we characterised the relationship between the expression of ataxin-3 containing polyQ expansion and mitochondrial impairment. We hypothesise that longer polyQ repeat lengths exacerbate mitochondrial alterations, as evidenced by altered mitochondrial morphology, reduced protein levels of key mitochondrial proteins, and increased sensitivity to mitochondrial stressors. By elucidating these mechanisms, this work aims to enhance our understanding of mitochondrial contributions to MJD pathogenesis and provide a foundation for therapeutic interventions targeting mitochondrial pathways in polyQ diseases.

## RESULTS

### Proteomic analysis reveals differentially regulated pathways in the brain of WT and MJD mice

To analyse changes in protein expression levels and altered metabolic pathways in the brains of CMVMJD135 mice (hereafter MJD mice) compared to those in the brains of WT mice, we performed global proteomic analysis of male and female WT and MJD mice. Raw files were searched using Proteome Discoverer 2.4 against the *Mus musculus* UniProt database (taxon ID 10090, accessed on 11 November 2021) and the protein list was filtered (q-value<0.01, protein abundance cutoff of ≤−2-fold or ≥2-fold with an adjusted *P*-value<0.05) for Ingenuity Pathway Analysis (IPA). IPA revealed widespread dysregulation of several cellular pathways. Among these, the oxidative phosphorylation and BAG2 signalling pathways were the most significantly downregulated (Fig. 1A).

**Fig. 1.**
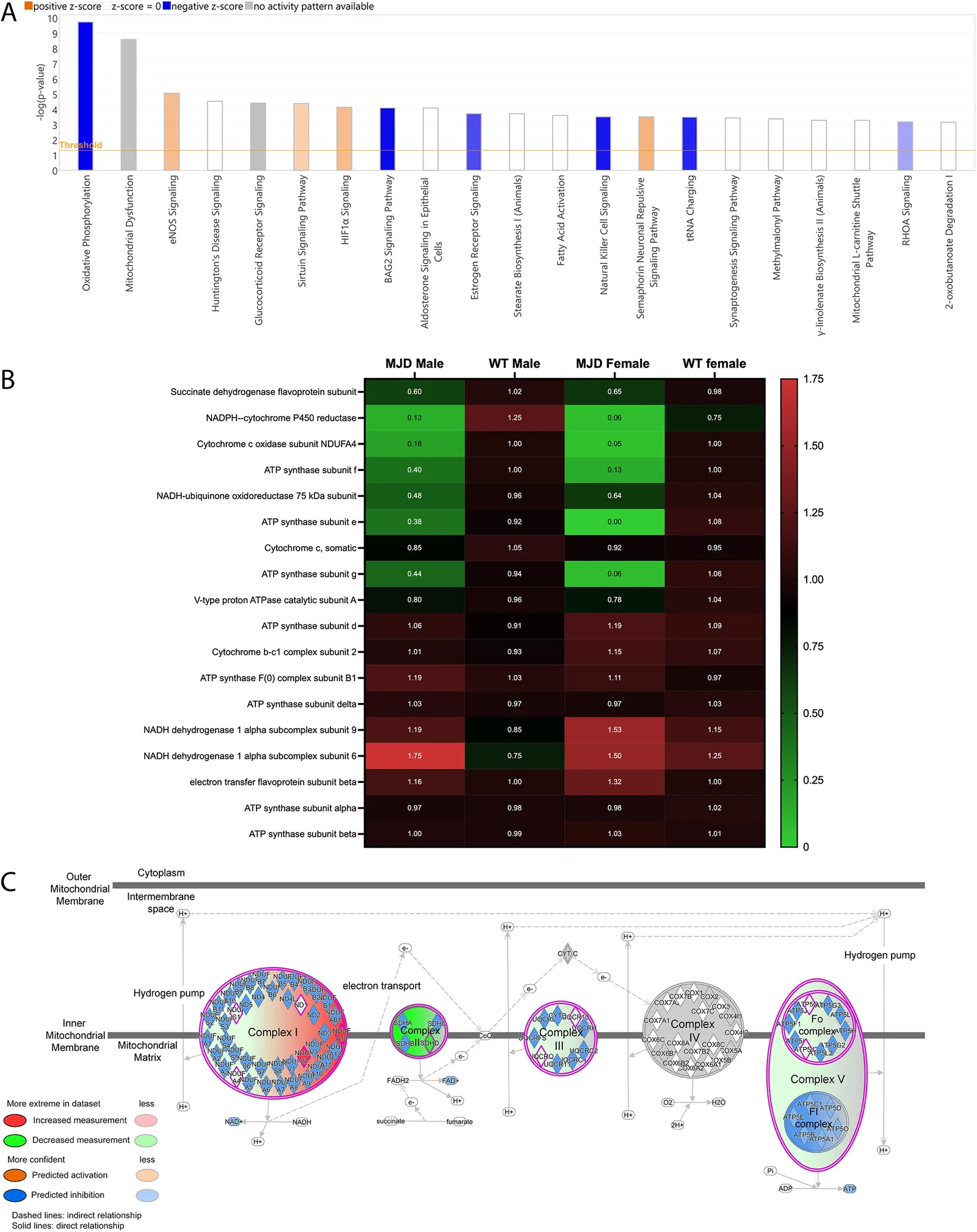
**Label-free quantitative proteomics of male and female CMVMJD135 transgenic mice and WT littermate controls reveals signs of mitochondrial impairment as a dominant feature.** (A) Ingenuity Pathway Analysis (IPA) performed on the male CMVMJD135 (MJD) versus wild-type (WT) littermate control dataset predicted an inhibition of oxidative phosphorylation and the BAG2 signalling pathway. Blue indicates IPA-predicted inhibition and orange indicates predicted activation of categorised biological function and canonical pathways. (B) Heatmap comparison of the abundance of various protein components of mitochondrial function, normalised to the mean WT control values (all male and female WT controls), reveals similar effects within the male and female CMVMJD135 mice. (C) IPA network display reveals that the male CMVMJD135 mice exhibit decreased abundance of particularly complex II, III and V subunits of the oxidative phosphorylation pathway.

Within the oxidative phosphorylation pathway, the most pronounced reductions were observed in complexes II (succinate dehydrogenase) and V (ATP synthase) (Fig. 1B). These complexes showed decreased expression of multiple subunits in MJD mouse brains compared to that in WT controls. Notably, these results were obtained from male mice; however, parallel analysis within the female cohort indicated similar mitochondrial dysfunction (Fig. S1A) and mitochondrial protein downregulation (Fig. 1B). This consistency across sexes suggests that mitochondrial and ETC alterations are a robust and sex-independent feature of the disease. The marked reduction of complex II, which links the tricarboxylic acid (TCA) cycle with the ETC, highlights downregulation of a key interface between central carbon metabolism and oxidative phosphorylation (Fig. 1C). Likewise, the downregulation of complex V components indicates reduced abundance of ATP synthase subunits.

### Western blot validation of proteomic findings in MJD neurons

To validate the proteomic results, we performed western blot analysis on proteins extracted from primary neurons derived from WT and MJD mice after 18 days *in vitro* (DIV) (Fig. 2A). As expected, neurons from MJD mice were found to express polyQ-expanded human ataxin-3 (Fig. 2G). Consistent with our proteomic data, we observed a significant reduction in expression of mtND5 (a subunit of complex I) (Fig. 2B,C) and SDHA (a subunit of complex II) (Fig. 2B,D), confirming deficiencies in key components of the ETC. Additionally, pyruvate kinase (PKM), a crucial enzyme that supports mitochondrial metabolism through glycolysis, was also decreased in MJD mouse primary neurons, suggesting broader impairments in mitochondrial metabolism (Fig. 2E). In contrast, VDAC1 was significantly upregulated in MJD mouse primary neurons (Fig. 2F). Given its role in regulating mitochondrial permeability and apoptosis (Tomasello et al., 2009), this increase in VDAC1 may be associated with mitochondrial dysfunction and elevated oxidative stress.

**Fig. 2.**
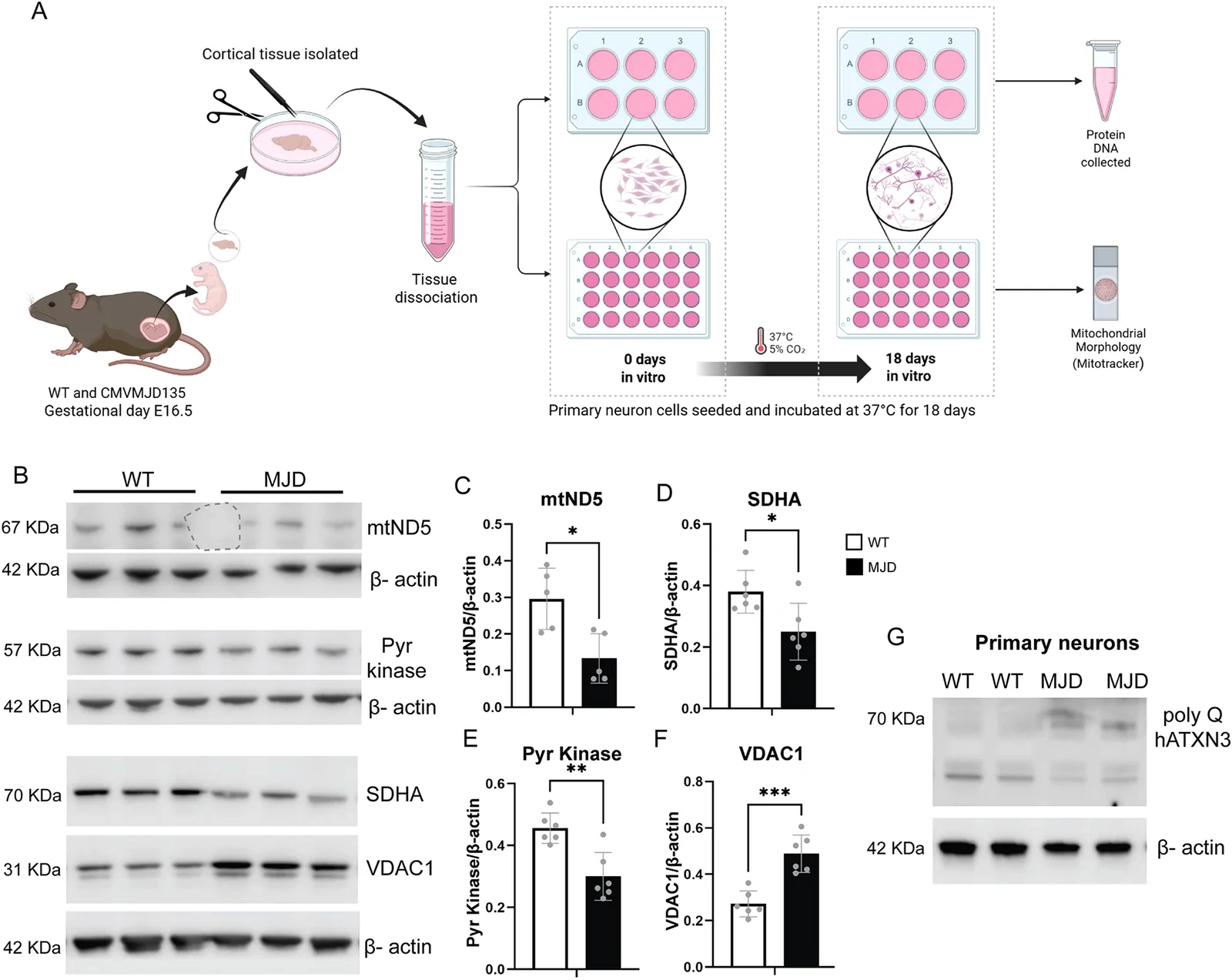
**Primary cortical neuron cultures from CMVMJD135 and WT embryos and analysis of mitochondrial and metabolic markers.** (A) Schematic overview of the primary neuron culture process. Cortical neurons were isolated from embryonic brains of pregnant CMVMJD135 and WT mice, cultured for 18 days *in vitro* (DIV), followed by protein or DNA extraction and MitoTracker analysis. Created in BioRender by Laird, A. S., 2026. https://BioRender.com/vfz6w29. This figure was sublicensed under CC-BY 4.0 terms. (B) Representative western blot images of *n*=2-3 samples per group for mtND5, pyruvate kinase, SDHA, VDAC1 and β-actin controls. Note that there was an image artefact affecting one WT and one MJD sample (demarcated with the dotted line), and these samples were not included in the analysis for this reason. (C-F) Quantification of mitochondrial and metabolic markers by western blotting in primary cortical neurons: (C) mtND5, (D) SDHA, (E) pyruvate kinase and (F) VDAC1. (G) Detection of mutant ATXN3 protein in primary neurons from MJD mice; ATXN3 was not detected in WT samples. The western blot images here show a subset of the samples; see full western blots in Fig. S4. β-actin was used as the loading control in all western blots. Data were obtained from six biological replicates, except for mtND5 (*n*=5). Bars show mean±s.e.m. Statistical comparisons involved two-tailed unpaired *t*-tests, and asterisks indicate statistically significant differences (*P<0.05; **P<0.01; ***P<0.001).

Western blotting of brain lysates of 20- to 25-week-old MJD mice for SDHA, mtND5, pyruvate kinase and VDAC1, and of spinal cord lysates from 16-week-old MJD mice for SDHA, mtND5, RISP (also known as UQCRFS1) and VDAC1 did not show significant genotype-dependent differences (Fig. S2). The only finding within these investigations was that COX10 expression was significantly decreased in 16-week-old MJD mouse spinal cord lysates (Fig. S2A,F).

### Effect of mutant ATXN3 on mitochondrial morphology and biomass

Fluorescence microscopy and quantitative image analysis revealed significant alterations in mitochondrial morphology in MJD primary neurons at 18 DIV compared to that in WT neurons (Fig. 3A). Specifically, mitochondria in MJD primary neurons exhibited a significant reduction in overall size and structural complexity compared to those in WT neurons. The average mitochondrial area significantly decreased from 0.259 μm^2^ in WT to 0.181 μm^2^ in MJD neurons, whereas the perimeter significantly decreased from 2.483 μm to 1.915 μm (Fig. 3B,C). Structural complexity, assessed by form factor, significantly dropped from 2.031 in WT to 1.718 in MJD, whereas the aspect ratio was not significantly different between the groups (Fig. 3D,L). Branching analysis revealed significant reductions in the number of branches (from 1.398 to 1.130), total branch length (from 0.780 μm to 0.495 μm), mean branch length (from 0.524 μm to 0.432 μm), number of branch junctions (from 0.211 to 0.069), number of branch endpoints (from 2.111 to 2.007) and longest shortest path (from 0.709 μm to 0.479 μm) in MJD neurons compared to those in WT neurons, respectively, whereas the mean branch diameter was not significantly different (Fig. 3E-K). These data demonstrate pronounced mitochondrial fragmentation and reduced network complexity in MJD primary mouse neurons.

**Fig. 3.**
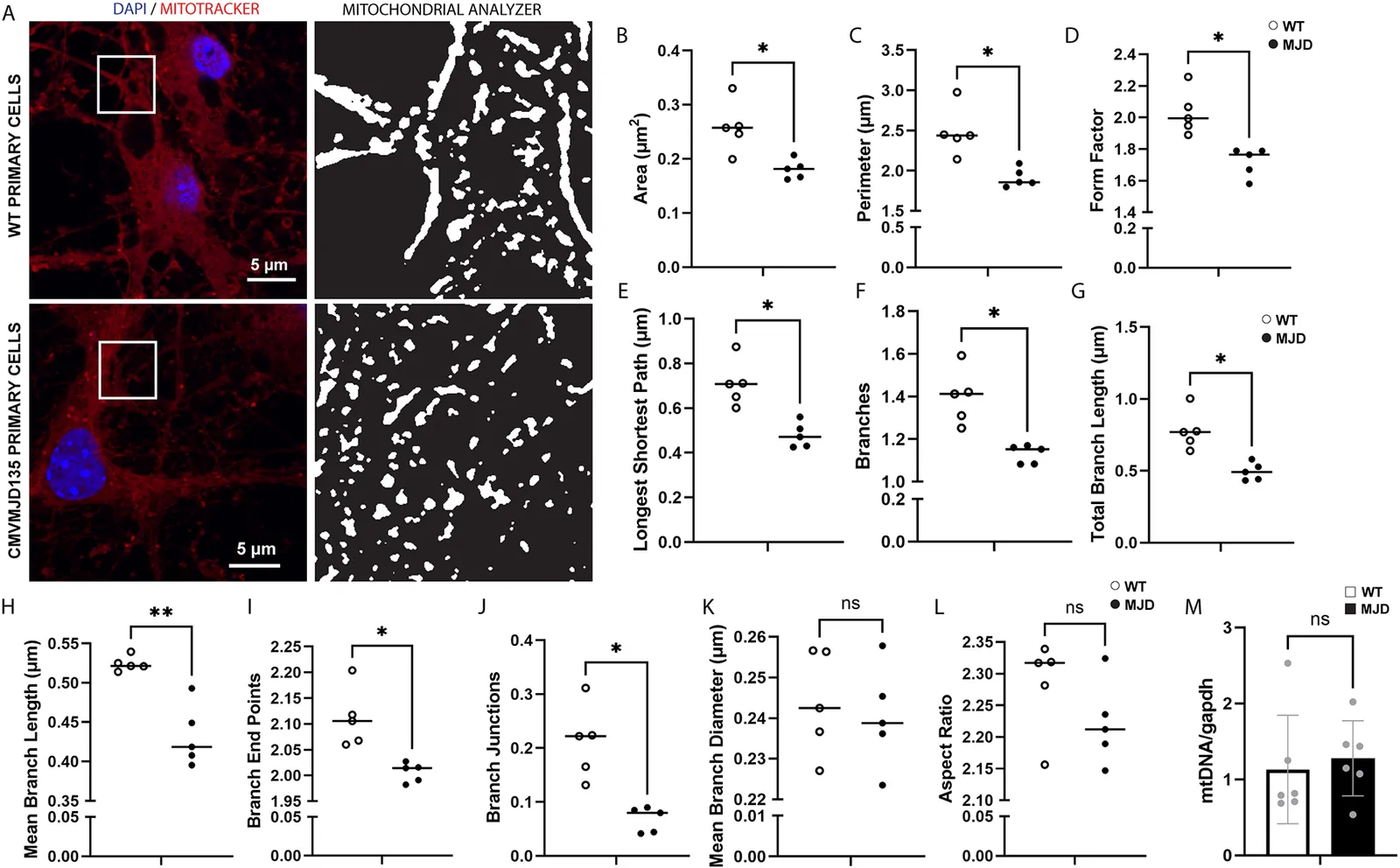
**Analysis of mitochondrial morphology and mtDNA content in primary cortical neurons from CMVMJD135 and WT mice.** (A) Representative confocal images of primary cortical neurons stained with MitoTracker Red (mitochondria) and DAPI (nuclei). Images include processed outputs from the mitochondrial morphology analyzer in ImageJ. (B-L) Quantification of mitochondrial morphology parameters in WT and MJD neurons: (B) area, (C) perimeter, (D) form factor, (E) longest shortest path, (F) number of branches, (G) total branch length, (H) mean branch length, (I) number of branch endpoints, (J) number of branch junctions, (K) mean branch diameter and (L) aspect ratio. (M) Mitochondrial DNA (mtDNA) quantification was performed by quantitative PCR. *Gapdh* was used as the nuclear reference gene. All analyses were performed using six biological replicates per group (WT and MJD). Bars show mean±s.e.m. Statistical comparisons involved two-tailed paired *t*-tests, and asterisks indicate statistically significant differences (ns, not significant; *P<0.05; **P<0.01).

To evaluate whether mitochondrial number was affected, mitochondrial DNA (mtDNA) levels were quantified by quantitative PCR (qPCR) using *Gapdh* as a reference gene. No significant differences in mtDNA levels were observed between MJD and WT neurons, suggesting that the mitochondrial biomass remains unchanged in disease (Fig. 3M). This indicates that the observed changes in mitochondrial morphology are not due to alterations in mitochondrial quantity but are likely a reflection of compromised mitochondrial quality or function.

### Genotype-dependent sensitivity of hATXN3 23Q and hATXN3 84Q zebrafish embryos to ETC disruptions

Next, we assessed the effects of ETC perturbation on transgenic zebrafish embryos expressing EGFP-fused human ataxin-3 (hATXN3) containing either 23 glutamines (23Q; WT protein) or 84 glutamines (84Q; MJD-causing) in neurons (driven by elav/HuC promoter). To do so, we administered a selective complex I inhibitor (rotenone, 30 nM) or DMSO (control) to hATXN3 23Q and hATXN3 84Q zebrafish embryos, as well as hATXN3 84Q-negative siblings (no EGFP-hATXN3 expression).

After 2 days of incubation in rotenone, hATXN3 84Q embryos exhibited a marked impairment, characterised by a decreased percentage of embryos exhibiting a response to tactile stimulation, whereas hATXN3 23Q and hATXN3 84Q-negative siblings remained unaffected (100% responsive, Fig. 4A). Importantly, no impairment was observed in the hATXN3 23Q or hATXN3 84Q-negative sibling groups, indicating that the observed phenotype was specific to hATXN3 84Q embryos.

**Fig. 4.**
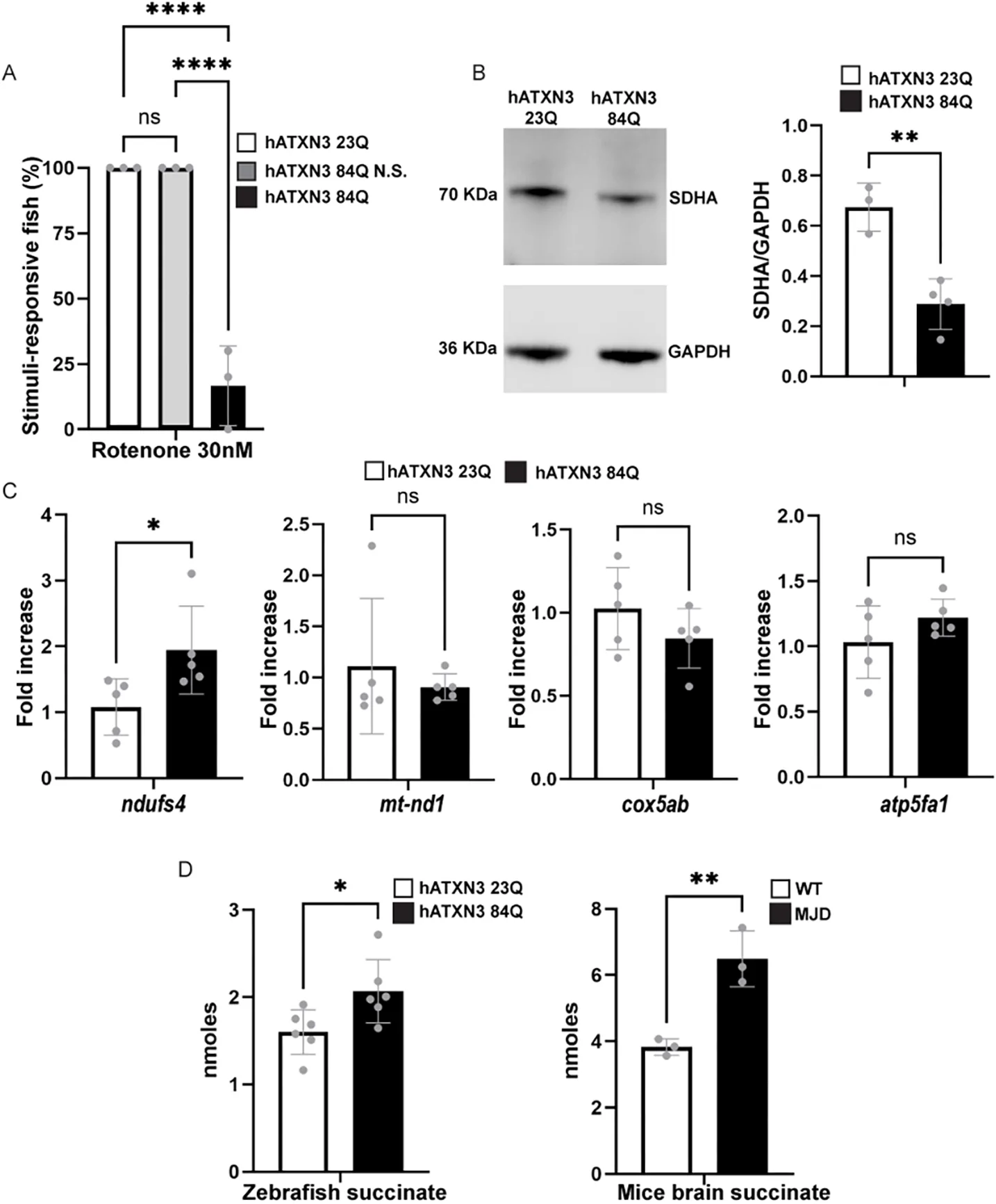
**Impact of polyQ-expanded ataxin-3 on mitochondrial function and succinate accumulation in zebrafish embryos and in mouse brains.** (A) Percentage of zebrafish embryos at 5 days post fertilisation (dpf) responding to stimuli following treatment (48 h) with rotenone (30 nM). White bars indicate hATXN3 23Q, grey bars represent hATXN3 84Q-negative siblings (N.S.), and black bars represent hATXN3 84Q embryos. (B) Western blot analysis of Sdha levels in hATXN3 23Q and 84Q zebrafish at 5 dpf. Gapdh was used as the loading control (*n*=3 for hATXN 23Q embryos; *n*=4 for hATXN3 84Q embryos). (C) Fold change in transcript levels of the mitochondrial genes *ndufs4*, *mtND1*, *cox5ab* and *atp5a1* in hATXN3 23Q and 84Q zebrafish at 5 dpf, as determined by quantitative PCR. *gapdh* was used as the reference gene (*n*=5 per group). (D) Quantification of succinate levels (nmol) in hATXN3 23Q and 84Q zebrafish (*n*=6 per group), and in WT and CMVMJD135 15-week-old mouse brains (*n*=3 per group). White bars represent 5 dpf hATXN3 23Q zebrafish and WT mouse samples, and dark bars represent hATXN3 84Q zebrafish and CMVMJD135 mouse samples. Bars show mean±s.e.m. Statistical comparisons involved two-tailed unpaired *t*-tests, and asterisks indicate statistically significant differences (ns, not significant; *P<0.05; **P<0.01; ****P<0.0001).

These findings indicate a genotype-dependent sensitivity to complex I inhibition, where hATXN3 84Q embryos display heightened vulnerability relative to hATXN3 23Q and hATXN3 84Q-negative sibling controls. Together, these data suggest that the pathogenic hATXN3 background lowers the threshold for ETC stress, resulting in impaired motor responsiveness upon complex I inhibition.

### Impaired complex II function and succinate accumulation in hATXN3 84Q zebrafish and MJD mice

To explore the molecular basis for the heightened sensitivity of hATXN3 84Q zebrafish embryos to rotenone, we evaluated the levels of Sdha, a subunit of complex II in the ETC, using western blot analysis (Fig. 4B). At 5 days post fertilisation (dpf), hATXN3 84Q zebrafish displayed a significant reduction in Sdha protein levels compared to hATXN3 23Q zebrafish (Fig. 4C). This decrease in complex II abundance suggests an impaired capacity to funnel electrons into the ETC through succinate oxidation. We also performed western blot analysis for other subunits, including CoxI (also known as Mt-co1), CoxIV (Cox4i1) and the mitochondrial protein Vdac1, finding no significant differences between hATXN3 23Q and hATXN3 84Q zebrafish lysates, except for CoxI, which was significantly elevated in hATXN3 84Q zebrafish (Fig. S3).

To further assess mitochondrial gene expression changes at the transcriptional level, we analysed the expression of key ETC-associated genes, including *ndufs4* (complex I), *mt-nd1* (complex I), *cox5ab* (complex IV), and *atp5a1* (complex V). hATXN3 84Q zebrafish exhibited a significant upregulation of *ndufs4* compared to its expression in hATXN3 23Q zebrafish, whereas no differences were observed for *mt-nd1*, *cox5ab* or *atp5a1* (Fig. 4C). The selective increase in *ndufs4* (a catalytic subunit) suggests a potential compensatory response of complex I to mitochondrial stress in the hATXN3 84Q background, in contrast to the stable expression of genes linked to complexes IV and V.

Additionally, we quantified succinate levels in zebrafish and mice models to assess the functional consequences of reduced SDHA protein. Succinate levels were significantly increased in both MJD zebrafish and MJD mouse brain samples compared to their respective WT controls (Fig. 4D). For zebrafish, the succinate levels were 1.601 nmol in hATXN3 23Q embryos and 2.068 nmol in hATXN3 84Q embryos, representing an increase in succinate accumulation in fish expressing longer polyQ expansions. This trend highlights the impact of the h*ATXN3* mutation severity on mitochondrial function. Similarly, in mouse brain samples, succinate levels were markedly higher in the MJD group compared to those in WT controls. WT mice exhibited succinate levels of 3.827 nmol, whereas MJD mice showed nearly double the amount at 6.491 nmol. These results confirm a significant disruption in mitochondrial metabolism associated with the disease phenotype in both zebrafish and mouse models.

This significant increase in succinate levels relative to WT controls is consistent with compromised activity of complex II. The observed succinate accumulation highlights a disruption in mitochondrial metabolic balance, which may exacerbate mitochondrial dysfunction in mutant zebrafish.

## DISCUSSION

### Mitochondrial pathway dysregulation in MJD mouse brains

To examine alterations to protein expression profiles in MJD brain tissue, we conducted proteomic profiling of WT and CMVMJD135 mice (20 to 25 weeks of age). Our analysis revealed extensive dysregulation of mitochondrial-associated pathways, with oxidative phosphorylation emerging as one of the most significantly affected processes, consistent with previous reports identifying mitochondrial bioenergetic deficits in polyQ disorders such as HD and spinocerebellar ataxias (Park et al., 2006). Similar metabolic pathway disruption has been described in MJD knock-in mouse models, in which TCA and oxidative phosphorylation-associated proteins are dysregulated in the cerebral cortex (Almeida et al., 2023). This dysregulation was also observed to increase with age, with metabolism-related proteins accounting for approximately 29% of the dysregulated proteins in the cerebellum of 4- to 10-month-old mice and 36% in 12- to 14-month-old mice, consistent with an age-dependent increase in metabolic dysregulation (Wiatr et al., 2021).

Within the brain proteomics, the ETC components showing the most prominent reductions were complexes II and V, with decreased abundance of multiple subunits. Notably, complex II (succinate dehydrogenase) is a critical bridge between the TCA cycle and the ETC; alterations in complex II have previously been associated with neurodegeneration and elevated reactive oxygen species (ROS) generation (Zheng et al., 2023; Van Vranken et al., 2014; Jodeiri Farshbaf and Kiani-Esfahani, 2018; Laço et al., 2012). In parallel, reduced abundance of ATP synthase (complex V) subunits is consistent with impaired energy synthesis, echoing findings in fibroblasts from patients with MJD and HD models (Harmuth et al., 2022).

Importantly, we identified similar proteomic changes across male and female cohorts of MJD mice, supporting a hypothesis that mitochondrial impairment is a robust and sex-independent hallmark of MJD pathogenesis. This finding is significant because previous studies by Almeida et al. (2023) have similarly found mitochondrial alterations to be present in male CMVMJD135 mice, but female CMVMJD135 mice were not examined.

### Neuron-intrinsic mitochondrial impairment in primary cortical neurons

Because bulk brain proteomic analyses reflect mixed cell populations, we next asked whether mitochondrial alterations are detectable directly in neurons. In primary cortical neuron cultures derived from CMVMJD135 embryos, western blotting revealed significant reductions in mtND5 (complex I) and SDHA (complex II), supporting neuron-intrinsic ETC alterations in the presence of mutant ataxin-3. Reduced mtND5 aligns with evidence that complex I disruption contributes to oxidative stress and vulnerability in HD (Parker et al., 1990) and in PD models (Dias et al., 2013). Reduced SDHA is consistent with constrained succinate oxidation and stress signalling linked to succinate accumulation (Siebels and Dröse, 2013; Kluckova et al., 2015).

We also detected pyruvate kinase downregulation, suggesting reduced substrate supply for mitochondrial metabolism. This finding is consistent with the impaired neuronal oxidative glucose metabolism reported in other neurodegenerative diseases, including Alzheimer's disease, in which pyruvate dehydrogenase, which catalyses the mitochondrial conversion of pyruvate to acetyl coenzyme A, has been found to be reduced, contributing to mitochondrial alterations (Bubber et al., 2005). In contrast, VDAC1 was increased in MJD neurons. VDAC1 regulates mitochondrial membrane permeability and has been associated with apoptosis driven by lipid peroxidation and ROS accumulation in neurodegenerative disease models (Shoshan-Barmatz et al., 2018). The upregulation of VDAC1 in MJD neurons may therefore reflect mitochondrial vulnerability to oxidative damage, linking mitochondrial alterations to apoptotic pathways in disease progression. Elevated VDAC1 expression has previously been reported in Alzheimer's disease, in which it correlates with increased oxidative stress (Reddy, 2013; Yang et al., 2024). VDAC1 also influences lipid peroxidation, a key feature of ferroptosis, a form of iron-dependent cell death (Zhao et al., 2020). The increased expression of VDAC1 in MJD neurons may reflect a mitochondrial stress response feedback loop, where oxidative stress exacerbates mitochondrial damage, further promoting cell death pathways such as ferroptosis.

Importantly, our proteomic analysis of brain tissue from MJD mice revealed significant downregulation of mitochondrial ETC components, a pattern consistent with reduced oxidative phosphorylation capacity and, potentially, diminished ATP production and respiratory function. Although these mice express mutant h*ATXN3* under the control of the ubiquitous CMV promoter, and the proteomic analysis thus reflects global protein expression changes, we strengthened these findings by validating mitochondrial protein alterations in a cell type-specific context using primary cortical neurons. This approach provides crucial additional value: it demonstrates that mitochondrial deficits occur directly in neurons, the primary affected cell type in MJD, rather than being secondary to systemic or glial changes. This cell type-specific validation confirms that the observed proteomic alterations are not only robust but also neutrally intrinsic, supporting the hypothesis that mutant h*ATXN3* directly disrupts neuronal mitochondrial function.

### Abnormal mitochondrial morphology in MJD mice-derived primary neurons

Quantitative fluorescence microscopy revealed mitochondrial fragmentation and reduced network complexity in MJD neurons at 18 DIV. Decreased mitochondrial size, branching and form factor are consistent with previous observations in HD, where mutant huntingtin impairs mitochondrial fusion via downregulation of MFN1/MFN2 and OPA1 (Shirendeb et al., 2011). Similar fragmentation patterns have been reported in MJD models, where mutant *ATXN3* disrupts mitochondrial dynamics and mitophagy (Hsu et al., 2017).

Although overall mitochondrial number (as assessed by mtDNA qPCR) remained unchanged, the observed morphological disruptions point to compromised mitochondrial quality rather than quantity. This correlates with studies in a human neuroblastoma cell line that indicated no significant changes in the amount of mitochondrial biomass in cells expressing the WT (26Q) and expanded (78Q) *ATXN3* gene (Chang et al., 2016). This distinction is important, as recent studies have shown that mitochondrial fragmentation often precedes energetic failure and neurodegeneration in polyQ disorders (Swinter et al., 2023).

### Cross-model validation and interpretation of partial concordance across assays, ages and tissues

We next integrated the discovery proteomics with targeted follow-up assays across additional contexts. Across these assays, complex II/SDHA emerged as the most consistent alteration across our platforms, spanning the discovery brain proteomics as well as targeted validation assays. In the proteomics dataset, SDHA was among the most prominently reduced features within the oxidative phosphorylation pathway, and this directionality was recapitulated by reduced SDHA detected in primary cortical neurons and in hATXN3 84Q zebrafish. This cross-model SDHA/complex II convergence aligns with succinate accumulation measured in both hATXN3 84Q zebrafish and the MJD mouse brain, supporting complex II-linked disruption as a shared feature even when other individual markers vary by tissue, age and assay modality. Although several other mitochondrial markers showed context-dependent differences, this recurring complex II signature provides a clear cross-model point of convergence.

In 5 dpf zebrafish expressing human *ATXN3*, CoxI was increased, whereas CoxIV and Vdac1 were not significantly altered. The selective increase in CoxI, in the absence of changes in CoxIV, may reflect stoichiometric remodelling and/or compensatory regulation of individual respiratory chain subunits rather than a uniform upregulation of complex IV. Likewise, reduced Sdha (a core complex II subunit) is consistent with a defect more aligned with complex II subunit abundance and/or stability. In mice, COX10 was significantly reduced in 16-week-old mice spinal cords, whereas mtND5, SDHA, RISP and VDAC1 levels were unchanged; in 20- to 25-week-old week brain lysates (the same age as the discovery proteomics cohort), none of the tested targets (mtND5, SDHA, pyruvate kinase, COX10 and VDAC1) differed significantly between genotypes, as detected by western blotting. Because COX10 functions in complex IV biogenesis (heme A synthesis/COX assembly) (Antonicka et al., 2003), its selective decrease in spinal cords may indicate region-specific vulnerability affecting complex IV assembly capacity, even when other complex IV markers are unchanged in bulk lysates.

We note that quantifying mitochondrial proteins by immunoblotting in whole-tissue homogenates can be technically challenging, particularly for membrane-associated ETC components, due to cellular heterogeneity, variable solubilisation of mitochondrial membranes, epitope accessibility and the limited sensitivity or dynamic range of antibody-based detection. These technical constraints, together with tissue composition and disease-stage differences, likely contribute to the partial concordance between discovery proteomics (whole brain) and targeted immunoblots (whole brain versus spinal cord) and help explain why some signals are more readily detected in neuron-enriched cultures and in zebrafish larvae than in adult bulk tissue. In this context, the agreement between discovery proteomics (complex II/SDHA downregulation) and orthogonal follow-up assays (reduced SDHA in neurons and zebrafish) strengthens the inference that complex II disruption is not assay specific but reflects a reproducible disease-associated feature. As a result, neuron-enriched preparations (primary neurons) and/or mitochondrial-enriched fractions are expected to provide more sensitive and interpretable readouts of mitochondrial alterations than bulk tissue lysates. Consistent with this, primary cortical neurons robustly recapitulated key ETC-associated changes and revealed pronounced mitochondrial network fragmentation, supporting neuron-intrinsic mitochondrial remodelling in MJD.

In contrast, complex I-associated readouts varied across models: NDUFA4 abundance was increased in MJD mouse brain proteomics (Fig. 1B), *ndufs4* mRNA was elevated in hATXN3 84Q zebrafish, and mtND5 was decreased in MJD primary neurons, whereas mtND5 expression did not differ in 16-week-old spinal cord lysates or 20- to 25-week-old week brain lysates by western blotting. This pattern may reflect context-dependent remodelling of complex I and/or differences between transcripts, protein abundance and neuron-enriched versus bulk-tissue measurements. For example, transcriptional upregulation (such as that of *ndufs4*) may represent compensation that is not matched at the protein level if mitochondrial import, turnover or complex assembly is altered.

### Complex II-linked metabolic disruption and ETC stress sensitivity

Despite tissue- and stage-dependent variation in individual protein markers, metabolite-level measures pointed to a convergent disruption of mitochondrial metabolism. Succinate was elevated in both MJD mouse brains and hATXN3 84Q zebrafish. Given that succinate is the substrate of complex II (succinate dehydrogenase or SDH), this is consistent with a bottleneck in succinate utilisation and/or complex II-linked flux. These findings are highly consistent with the literature; mutations or inhibition of different SDH subunits have been shown to result in succinate buildup, which can act as an oncometabolite and pro-inflammatory signal (Armstrong et al., 2022; Eijkelenkamp et al., 2020; Bardella et al., 2011). In neurological disease models, elevated succinate has been linked to inflammation, impaired mitochondrial respiration, increased ROS generation and stabilisation of HIF-1α under normoxic conditions, exacerbating neurodegenerative phenotypes (Zhang et al., 2020; Huang et al., 2024; Tretter et al., 2016; Gonzalez-Meler et al., 1996; Scialò et al., 2017, 2016; Li et al., 2020).

In line with heightened ETC vulnerability, zebrafish embryos expressing hATXN3 84Q displayed heightened sensitivity to rotenone (complex I inhibitor), suggesting a specific vulnerability to ETC disruptions. Complex I, which plays a critical role in transferring electrons from NADH to the ubiquinone pool, is an essential entry point for mitochondrial respiration and ATP synthesis. Increased sensitivity to complex I inhibition in hATXN3 84Q embryos may point to disruptions in mitochondrial metabolic pathways, such as altered NADH turnover or reduced complex I activity. These findings align with studies in HD zebrafish and *Drosophila melanogaster* models, in which rotenone exposure induces developmental abnormalities, ROS accumulation and early lethality due to compromised complex I activity (Wang et al., 2017; Coulom and Birman, 2004).

### Timing of metabolic disruption and implications for therapeutic targeting

It is important to note that proteomic data were obtained from MJD mouse brains at 20-25 weeks of age, whereas succinate quantification were performed in an independent cohort euthanised at 15 weeks, to allow identification of alterations present at an earlier disease stage and therefore therapeutically targetable age. The observation of elevated succinate at this earlier stage suggests that metabolic alterations at complex II may precede and potentially contribute to the broader proteomic and functional mitochondrial changes detected at later stages of disease progression. The increase in succinate observed in MJD mouse brains suggests a metabolic bottleneck at complex II, which likely contributes to energy deficits and oxidative stress. When combined with rotenone treatment (complex I inhibition) in the hATXN3 84Q zebrafish embryos, the primary entry point for electrons into the ETC is blocked. In the context of reduced SDHA expression and impaired complex II function, the electron flow into the ETC is further diminished, severely limiting mitochondrial respiration and energy production. The combined inhibition of complex I by rotenone and reduced complex II capacity in the MJD zebrafish likely explains the pronounced developmental delay observed in hATXN3 84Q embryos following rotenone exposure. This suggests a critical interplay between ETC components in maintaining mitochondrial function in disease models.

The mitochondrial deficits identified here suggest several therapeutic avenues worthy of future investigation: (1) mitochondria-targeted antioxidants such as MitoQ, which can counter the ROS generation and lipid peroxidation that follow complex II dysregulation (McManus et al., 2011); (2) enhancement of PINK1/parkin-mediated mitophagy to promote clearance of the fragmented mitochondria and elevated VDAC1 expression observed in MJD neurons (Yang et al., 2021); and (3) NAD^+^ precursor supplementation to broadly support oxidative phosphorylation capacity (Yang et al., 2021). Notably, our finding that succinate accumulation occurs early in the CMVMJD135 mouse model suggests that early intervention may be critical to the efficacy of any such approach.

### Conclusions

In summary, this study adds to growing evidence that polyQ-expanded ataxin-3 is associated with mitochondrial alterations across experimental models of MJD. Although individual ETC markers showed region- and stage-dependent changes across validation contexts, convergent evidence from proteomics, neuronal cultures, zebrafish assays and succinate measurements points to complex II-linked metabolic disruption as a consistent feature. We further stablished MJD zebrafish and CMVMJD135-derived primary neurons as complementary, tractable platforms for mechanistic studies. Importantly, identifying the specific mitochondrial pathways disrupted by mutant ataxin-3 may guide the development of targeted interventions designed to restore mitochondrial homeostasis and neuronal viability. Further studies are needed to confirm the presence of these functional changes in human cells and to define their underlying mechanisms.

## MATERIALS AND METHODS

### Care and use of animals

All animal experiments were conducted in accordance with the Australian Code for the Care and Use of Animals for Scientific purposes (8th Edition, 2013) and approved by the animal ethics committee of Macquarie University (Animal Research Authorities: 2016/004, 2017/044, 2020/028, 2017/030 and 2017/019).

### Mouse breeding

Transgenic CMVMJD135 mice expressing mutant human *ATXN3* with 135 CAG repeats under a CMV promoter were bred and housed at Australian BioResources (Moss Vale, Australia) under pathogen-free conditions. Transgenic mice expressing human mutant *ATXN3* (CMVMJD135 mice) were bred with wild-type C57BL/6 mice to obtain heterozygous *ATXN3*-expressing mice (MJD) and non-transgenic littermate controls (WT). Transgene expression was confirmed via PCR, and inherited CAG repeat expansion length in MJD animals was confirmed via fragment analysis.

This study utilised two independent cohorts of CMVMJD135 and non-transgenic littermate mice. The first cohort, previously described by Gamage et al. (2023), was used for proteomic analyses. Briefly, animals were delivered to the Central Animal Facility at Macquarie University and allowed to acclimate for 7 days prior to commencing experimentation. Animals were group-housed according to sex and genotype in exhaust-ventilated cages with *ad libitum* access to standard chow and water. Mice were provided red domes or cardboard tunnels for environmental enrichment, and housing rooms were maintained at 21°C with a 12-h/12-h light/dark cycle (lights on at 06:00). All animals underwent weekly monitoring to assess wellbeing and neurological symptoms. The presence or absence of neurological symptoms such as hindlimb reflex, tremor and gait abnormalities were scored on a four-point scale, with a score of 0 indicating absence of neurological symptoms and a score of 4 indicating severe neurological symptoms. CMVMJD135 mice were deemed to reach humane endpoints upon reaching a total neurological score of 10, and were euthanised (age range: 20-25 weeks). Non-transgenic littermates were euthanised at 25 weeks of age. Mice were euthanised via intraperitoneal injection of sodium pentabarbitone (300 mg/kg). Once deeply anaesthetised, intracardiac perfusion was performed with ice-cold phosphate buffer saline (PBS). Brains were extracted and hemisected. One hemisphere of the brain was snap frozen in liquid nitrogen and stored at −80°C until processing. Brain tissue from a total of 20 mice (*n*=5 male MJD, 5 male WT, 5 female MJD, 5 female WT) were used for proteomic experiments.

The second cohort was generated and maintained under identical housing and monitoring conditions but was used for succinate quantification. In this cohort, mice (*n*=3 male MJD, 3 male WT) were euthanised at 15 weeks of age, and whole brains were rapidly collected, snap frozen in liquid nitrogen and stored at −80°C for subsequent succinate extraction and analysis.

For further analysis of relevant protein abundances, we performed western blotting on lysates extracted from the first male cohort mice (20-25 weeks of age) with an additional four mice per genotype, taking those comparisons to *n*=9 male MJD and 9 male WT mice. Additionally, we performed western blot analysis of spinal cord protein lysates extracted from 16-week-old mice (*n*=5 each for male MJD and male WT).

### Primary neuron culture

Pregnant female mice were euthanised via cervical dislocation on gestational day 16 (E16). Primary neuronal cultures were prepared as per Fath et al. (2009). Embryos were extracted and anaesthetised in ice-cold Hanks Buffered Saline Solution. The cortex was dissected from the brains of mixed gender and genotype embryonic mice at E16 and dissociated via mechanical trituration following exposure to trypsin and DNase I. Neurons were plated on poly-D-lysine-coated well plates. For protein and DNA extraction, 350,000 cells per well were seeded in six-well plates, whereas for MitoTracker staining, 50,000 cells per well were plated in 24-well plates containing glass coverslips. Initially, cells were cultured in Dulbecco's modified Eagle medium (DMEM, Gibco) supplemented with 10% fetal bovine serum (Gibco). 2 h post plating, the DMEM was aspirated and replaced with neurobasal medium (Gibco) containing 2% (v/v) B27 supplement (Gibco) and 0.25% (v/v) Glutamax (Gibco). Neurons were maintained in a 37°C incubator with 5% CO_2_. For primary neuron experiments, each biological replicate corresponded to an independent pooled neuronal preparation generated from multiple embryos. Where the same pooled preparation was plated across multiple wells, these wells were treated as technical replicates.

### Zebrafish maintenance

Zebrafish were maintained under standard conditions in a recirculating aquarium system at 28.5°C with a 13-h light/11-h dark cycle. Zebrafish embryos used in this study were obtained from transgenic MJD zebrafish generated as previously described (Watchon et al., 2017). This line was established by crossing the *Tg(elavl3:Gal4-VP16; mCherry)* driver line with responder lines *Tg(UAS:dsRED,EGFP-ATXN3_Q23)* or *Tg(UAS:dsRED,EGFP-ATXN3_Q84)*, allowing neuronal expression of human *ATXN3* (hATXN3 23Q or hATXN3 84Q) fused to EGFP. F1 zebrafish embryos were used for further analysis and were maintained in E3 medium under standard conditions throughout the experiments.

### Zebrafish drug treatment

EGFP-hATXN3-23Q, EGFP-hATXN3-84Q and EGFP-hATXN3-84Q-negative sibling (same genetic background as the EGFP-hATXN3-84Q but not showing GFP expression) zebrafish embryos were obtained from three independent matings and, at 3 dpf, randomly divided into two treatment groups: DMSO and 30 nM rotenone (Sigma-Aldrich, R8875; prepared in DMSO). Treatments were administered by adding the compounds directly to the E3 medium in which the embryos were housed and maintained continuously for 2 days. After this period, embryos were analysed and classified to assess their response to tactile stimulation by gently touching them with a 20 μl pipette tip.

### Protein extraction

For mouse cerebellar protein extraction, cerebellum tissue was dissected and placed in radioimmunoprecipitation (RIPA) buffer (5 μl/mg) containing cOmplete Protease Inhibitor Cocktail (Roche) and PhosSTOP Phosphatase Inhibitor Cocktail tablets (Roche). Cerebellar tissue was homogenised via sonication using an Omniruptor 250 Ultrasonic Homogeniser (Omni International).

For primary cell protein extraction, cells at 18 DIV were lysed in 30 μl per well of RIPA buffer containing cOmplete Protease Inhibitor Cocktail and PhosSTOP Phosphatase Inhibitor Cocktail tablets. After scraping the cells in one well, the lysates were transferred to the next new well and scraped again. Lysates were pooled for a total number of 600,000 cells per tube.

For zebrafish protein extraction, 20 zebrafish embryos (5 dpf) were used per replicate. The yolk was removed following the procedure described in Link et al. (2006). The remaining tissue was homogenised in RIPA buffer containing cOmplete Protease Inhibitor Cocktail and PhosSTOP Phosphatase Inhibitor Cocktail tablets.

In all cases, the homogenised samples were then centrifuged at 13,200 ***g*** or 14,000 ***g*** for 15-20 min at 4°C, and the supernatant containing the RIPA-soluble proteins was collected for analysis. The total protein concentration was determined using a Pierce BCA Protein Assay Kit (Thermo Fisher Scientific) according to the manufacturer's instructions.

### Western blotting

For western blotting, 20 μg of protein from each sample was prepared with NuPage Sample Reducing Agent (Invitrogen) containing dithiothreitol and Laemmli sample buffer (Bio-Rad), denatured, and separated using a 4-12% acrylamide gradient NuPAGE Bis-Tris gel (Invitrogen). Proteins from both primary cell and zebrafish samples were transferred to a 0.45 μm polyvinylidene difluoride (PVDF) membrane (Cytiva) and incubated overnight in TBS containing 0.1% Tween 20 and 5% BSA, with the following specific primary antibodies: anti-ATXN3 (1:20,000; kind gift from Henry Paulson, University of Michigan, Ann Arbor, MI, USA), β-actin (1:5000; Sigma-Aldrich, A5441), anti-mtND5 (1:2000; Abcam, ab92624), anti-SDHA (1:3000; Cell Signaling Technology, D6J9M), anti-VDAC1 (1:2000; Abcam, ab15895), anti-pyruvate kinase (1:2000; Abcam, ab156849), anti-RISP (1:1000, Cell Signaling Technology, 95231), anti-COX10 (1:1000, Cell Signaling Technology, 24744), anti-CoxI (1:1000, Cell Signaling Technology, 55159) and anti-CoxIV (1:1000, Cell Signaling Technology, 4850). The immunoblots were incubated with the corresponding secondary antibodies: goat anti-rabbit IgG (H+L) HRP conjugate (1:10,000, Promega, W4011) for rabbit primary antibodies, and goat anti-mouse IgG (H+L) HRP conjugate (1:10,000, Promega, W4021) for mouse primary antibodies. The protein bands were visualised using the ImageQuant LAS 4000 imager (Cytiva) by chemiluminescence reaction with Clarity Western ECL Substrate (Bio-Rad). The images were exported as digital 8-bit TIFF files and analysed using ImageStudio (LI-COR Biosciences). Quantification of the target proteins was performed, with normalisation to Gapdh (for zebrafish samples) or β-actin (for primary cell samples), which were used as loading controls. Blot transparency images are shown in Fig. S4. For densitometry of primary neuron western blots, each data point represents one independently generated pooled-embryo culture (biological replicate). When the same pooled-embryo culture was plated into multiple wells and processed in parallel, densitometry values from those wells were averaged to generate a single value per pool prior to statistical analysis. Where such technical replicate wells are shown in full membrane images in Fig. S4, they are indicated with letter suffixes (e.g. ‘a’ and ‘b’) to denote wells originating from the same pooled preparation.

### DNA extraction

Total DNA, including mtDNA, was extracted from primary cells cultured in six-well plates by adding 300 μl of TRIzol reagent (Invitrogen, 15596026) directly to the wells. DNA was isolated following the manufacturer's protocol, ensuring recovery of both nuclear DNA and mtDNA for downstream analysis.

### RNA extraction and cDNA synthesis

EGFP-hATXN3-23Q and EGFP-hATXN3-84Q zebrafish larvae (*n*=30 each, at 5 dpf) were suspended in TRIzol reagent and homogenised using a plastic pestle. Homogenised tissue samples were centrifuged at 15,000 ***g*** for 15 min, and the supernatant was transferred to a new tube. Total RNA was isolated following the manufacturer's protocol for TRIzol reagent. RNA samples were treated with RQ1 DNase I (Promega, M6101) for 30 min to remove DNA contamination. RNA purity and concentration were assessed using a NanoDrop spectrophotometer (Thermo Fisher Scientific). Reverse transcription of 5 μg of total RNA to cDNA was performed using the High-Capacity cDNA Reverse Transcription Kit (Thermo Fisher Scientific, 4368814) in a total reaction volume of 20 μl. The resulting cDNA was diluted to a final volume of 150 μl.

### qPCR

Primers for mouse samples (ms*Gapdh* and *mtDNA*) and for zebrafish (*ndufs4*, *mt-nd1*, *cox5ab*, *atp5fa1* and zf*gapdh*) were designed using Primer3web version 4.1.0 (https://primer3.ut.ee/) based on the sequences obtained from NCBI (Table S1). Prior to real-time reverse-transcription qPCR (RT-qPCR) analysis, the efficiency of amplification for all primer pairs was evaluated to ensure proper use of the ΔΔCT method. RT-qPCR reactions were performed on QuantStudio7 Pro (Applied Biosystems) in a 10 μl final volume composed of 1 μl of cDNA or DNA template, 5 μl of 2× SYBR Green master mix (Thermo Fisher Scientific, 4367659), 0.5 μl of 10 μM forward and reverse primer mix, and sterile water. Melting curve analysis was conducted to confirm the specificity of the real-time RT-qPCR reaction, which was programmed to include a melting profile immediately following the thermal cycling protocol. Mean cycle threshold (CT) values were used for ΔΔCT calculations. Fold differences in gene expression were calculated using the ΔΔCT method (Livak and Schmittgen, 2001) and gene expression levels were normalised to those of *Gapdh*. Stability of *Gapdh* expression was confirmed by comparing raw CT values between both genotypes: no differences were detected and CT values remained consistent within groups. The results are presented as the fold change of target gene expression relative to a reference sample (hATXN3 23Q) and normalised to the reference gene (*Gapdh*). All reactions were performed in duplicate and no-template controls were included to confirm the absence of contamination.

### Succinate quantification

Succinate concentrations were assessed by colorimetry using commercially available kits (Abcam, ab204718). Transgenic zebrafish expressing EGFP-hATXN3-23Q or EGFP-hATXN3-84Q were maintained at 28.5°C in standard E3 medium. At 5 dpf, pools of 50 embryos were prepared for each biological replicate, with six replicates analysed per genotype. Embryos were rinsed in extraction buffer and homogenised according to the instructions provided by the succinate assay kit manufacturer. Mouse brain samples were collected from WT and MJD mice belonging to the second cohort described above. In this cohort, animals were euthanised at 15 weeks of age, and whole brains were rapidly collected, snap frozen in liquid nitrogen, and stored at −80°C until analysis. For each condition, three biological replicates were used, with each replicate consisting of 100 mg of brain tissue. Brain samples were homogenised in the extraction buffer provided in the kit using a glass dounce homogeniser (Potter-Elvehjem) on ice and centrifuged at 10,000 ***g*** for 10 min to remove debris. The supernatants were collected and stored at −80°C for subsequent analysis. Homogenised zebrafish and mouse brain samples were deproteinised using 10 kDa molecular mass cutoff spin filters (Abcam, ab93349) to eliminate interfering proteins. The filtrates were diluted in the assay buffer provided in the kit to ensure that absorbance readings fell within the linear range of the standard curve. Succinate levels were measured by a colorimetric assay that detects succinate through a coupled enzymatic reaction producing a signal at 450 nm. A standard curve was prepared with succinate concentrations ranging from 0 to 10 nmol/well. 50 μl of each sample or standard was added to a 96-well plate in triplicate, and the reaction mix was prepared and added to each well per the manufacturer's instructions. Plates were incubated at 37°C for 30 min, protected from light, and the absorbance at 450 nm was measured using a CLARIOstar Plus Microplate Reader (BMG Labtech). Succinate concentrations were calculated from the standard curve using linear regression for zebrafish samples or tissue samples.

### MitoTracker staining

For mitochondrial staining, primary cells cultured on coverslips in 24-well plates at 18 DIV were incubated in 500 μl of MitoTracker Deep Red FM (Thermo Fisher Scientific, M22426) at a final concentration of 100 nM in neurobasal medium (Gibco, 21103049) without serum for 30 min at 37°C. Following incubation, cells were washed twice with 1× PBS and fixed for 15 min in 3.7% paraformaldehyde at room temperature. After two additional 1× PBS washes, coverslips were mounted with mounting medium with DAPI (Fluoroshield, Abcam, ab104139) for subsequent confocal microscopy analysis.

### In-gel digestion

Approximately 20 μg of brain protein lysates was digested for proteomics analysis as described previously (Cheng et al., 2021). In short, the lysates were boiled at 95°C for 5 min in 1× Laemmli loading buffer (Bio-Rad, 1610747) containing 1× reducing reagent (Invitrogen, NP0004). The lysates were run 3 cm into a 4-12% Bis-Tris SDS-PAGE gel (WG1402BOX, Invitrogen) and excised from the gel into three fractions. Proteins were reduced with 10 mM dithiothreitol (D0632, Sigma-Aldrich) for 30 min at 55°C, alkylated with 55 mM iodoacetamide (Sigma-Aldrich, I1149) in the dark for 45 min at room temperature, then digested with trypsin (1:50 enzyme:protein ratio, Thermo Fisher Scientific, 90057) overnight at 37°C. Formic acid (Sigma-Aldrich, 5330020050) was added to a final concentration of 1% to inactivate the digestion. The extracted tryptic peptides were desalted on a pre-equilibrated C_18_ Omix tip (Agilent Technologies, A57003100K) and eluted in 50% (v/v) acetonitrile (Sigma-Aldrich, 1000292500) in 0.1% formic acid and dried under vacuum centrifugation. Lyophilised peptides were resuspended in 0.1% formic acid for liquid chromatography (LC)-tandem mass spectrometry (MS/MS) analysis.

### Reverse-phase LC-MS/MS

The peptides were separated on an Ultimate 3000 nanoLC (Thermo Fisher Scientific) fitted with the Acclaim PepMap RSLC column (2 μm particle size, 0.075 mm diameter and 150 mm length, Thermo Fisher Scientific, 164534), using a 60 min gradient [2-80% (v/v) acetonitrile, 0.1% (v/v) formic acid] running at a flow rate of 300 nl/min. The electrospray source was fitted with a 10 μm emitter tip and maintained at 1.6 kV voltage. Peptides eluted from the nanoLC column were ionised into the Q-Exactive Plus mass spectrometer (Thermo Fisher Scientific). The full MS scan was carried out within the range of 400-2000 mass-to-charge ratio. Precursor ions were selected for MS/MS fragmentation using a data-dependent ‘top 10’ method operating in Fourier Transform (FT) acquisition mode with higher-energy C-trap dissociation (HCD) fragmentation. FT-MS analysis was carried out at 70,000 resolution and with an automated gain control (AGC) target of 1×10^6^ in full MS. MS/MS scans were carried out at 17,500 resolution with an AGC target of 2×10^4^ ions. Maximum injection times for full MS and MS/MS were set at 30 and 50 ms, respectively. The charge exclusion was set to unassigned and 1+ charged state with a dynamic exclusion of 20 s. The ion selection threshold for triggering MS/MS was set to 25,000 counts, and an isolation width of 2.0 Da was used to perform HCD fragmentation, with a normalised collision energy of 27.

### Data analysis

Raw spectra files were processed using Proteome Discoverer software 2.4 (Thermo Fisher Scientific) incorporating the SEQUEST search algorithm. Peptide identification was determined using a 20-ppm precursor ion tolerance and a 0.1 Da MS/MS fragment ion tolerance for FT-MS and HCD fragmentation. Carbamidomethylation (+57.021 Da) of cysteines as a static modification and oxidation (+15.995 Da) of methionine and N-terminal acetylation (+42.011 Da) as variable modifications were set with a maximum of two missed cleavages allowed. The data were processed using Percolator (The et al., 2016) to estimate false discovery rates (FDRs), with protein identifications validated at a q-value threshold of 0.01. Label-free quantification was performed using Minora feature detection in Proteome Discoverer 2.4, applying the default parameters for precursor ion quantification. Briefly, peptide spectral matches (PSMs) were filtered using a maximum ΔCn (delta correlation number) of 0.05, a rank of 0, and a Δmass of 0 ppm. PSM and peptide validation was carried out using a strict FDR of 0.01 and a relaxed FDR of 0.05. Peptides shorter than six amino acids were excluded from the analysis.

For each sample set, chromatographic alignment of PSMs was conducted with a mass tolerance of 10 ppm and a maximum retention time shift of 10 min. Peptide groups used in protein quantification followed default settings, where a peptide was considered unique if present in only one protein group. Quantification was based on both unique and razor peptides (those shared among multiple protein groups) using precursor ion intensity.

Protein abundance was determined by summing the abundances of individual peptide groups, and protein ratios were calculated using pairwise comparisons based on the geometric median of peptide group ratios. Statistical significance was assessed using two-tailed unpaired Student's *t*-test, comparing each peptide or protein against the background distribution of all ratio values. *P*-values were adjusted for multiple testing using Benjamini–Hochberg correction. The proteomics data have been deposited to the ProteomeXchange Consortium via the PRIDE (Perez-Riverol et al., 2022) partner repository with the dataset identifier PXD063591 (https://doi.org/10.6019/PXD063591).

### Image deconvolution, processing and thresholding

For MitoTracker image deconvolution, processing and thresholding, we utilised the Mitochondrial Analyzer plugin in ImageJ (Chaudhry et al., 2020). Briefly, mitochondrial morphology was analysed from two-dimensional images acquired using Leica Stellaris 5 (Leica) with a 63× oil-immersion objective lens using a 3× zoom at 1568×1568 pixels. Raw images were pre-processed to enhance mitochondrial signal and reduce background noise as follows: background subtraction (radius=1 μm) to minimise non-mitochondrial fluorescence; sigma filtering (radius=0.1 μm, sigma=2.0) to smooth signal while preserving edges; contrast enhancement (block size=64, slope=2.0) to improve visualisation of dim structures; and gamma correction (value=0.80) to normalise intensity across images. Mitochondria were segmented using adaptive thresholding (block size=1.25 μm, empirically determined C value, where C is the constant offset applied to the locally calculated threshold). Post-processing steps included despeckling, removal of outliers (radius=0.15 μm) and binary mask generation for final segmentation. Using the Mitochondrial Analyzer plugin, we extracted key parameters including area and perimeter, form factor and aspect ratio (to evaluate mitochondrial elongation and branching) and branching metrics (to quantify mitochondrial network complexity).

### Statistics

Data analysis was performed using GraphPad Prism (version 10) software. Group comparisons were made using one-way ANOVA, followed by a Tukey's post hoc test for statistical significance. In cases in which only two groups were compared, comparisons were made using two-tailed unpaired Student's *t*-test. All graphs display mean±s.e.m.

## Supplementary Material

10.1242/dmm.052757_sup1 Supplementary information
