# Supplementary material for: PolyQ-expanded ataxin-3 is associated with mitochondrial alterations in Machado–Joseph disease
Source: Dis Model Mech. 2026 Sep 18;19(9):dmm052757. doi: 10.1242/dmm.052757 (PMC13617979; doi:10.1242/dmm.052757)
Supplement: Supplementary file 1 [file dmm-19-052757-s1.pdf]

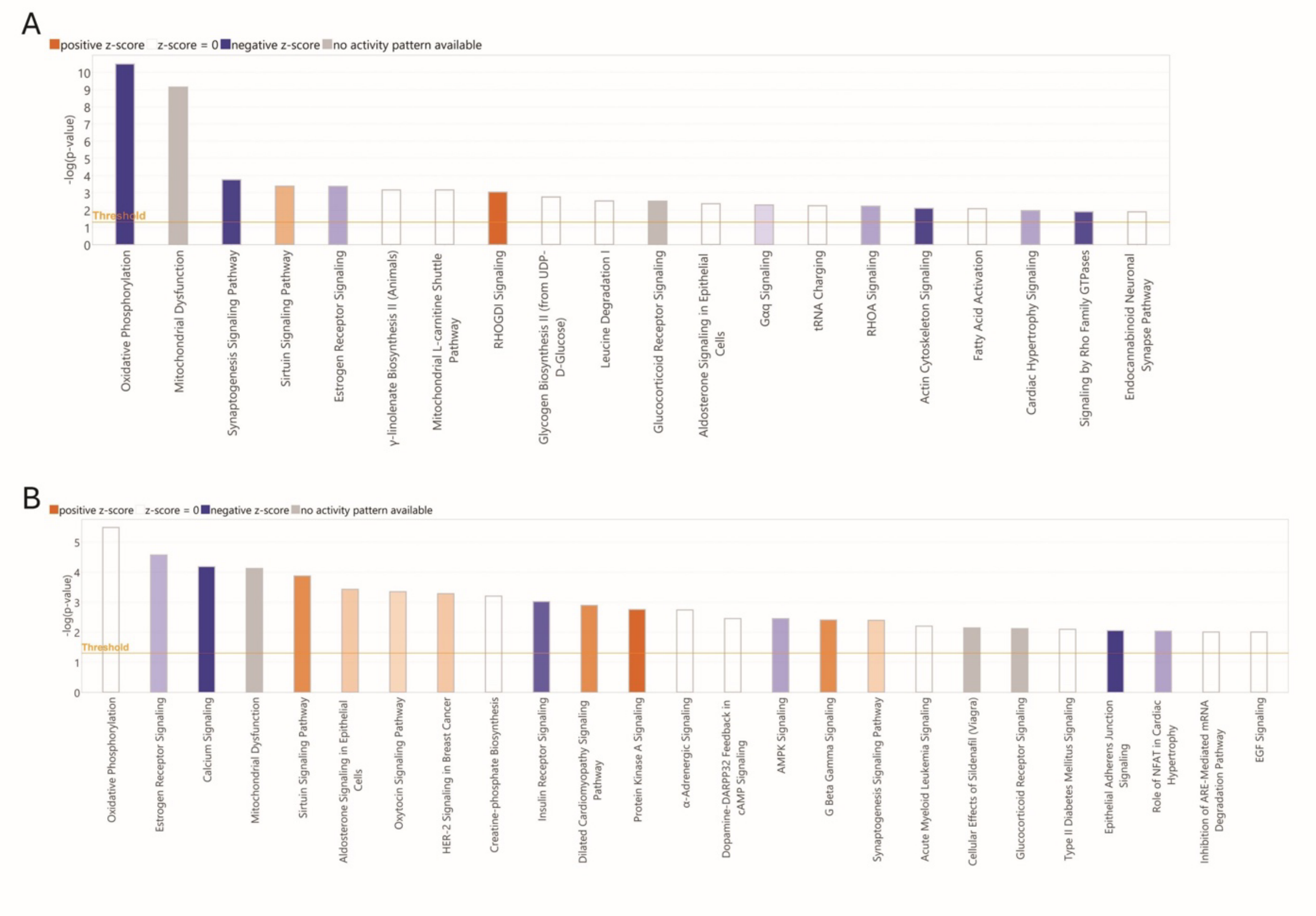

Fig. S1. Ingenuity Pathway Analysis (IPA) of oxidative phosphorylation in CMVMJD135 mice. (A) IPA predicted inhibition of oxidative phosphorylation in female MJD mice compared with WT littermate controls. (B) IPA did not identify differences in oxidative phosphorylation between male and female MJD mice. Blue indicates predicted inhibition and orange indicates predicted activation.

Disease Models & Mechanisms • Supplementary information

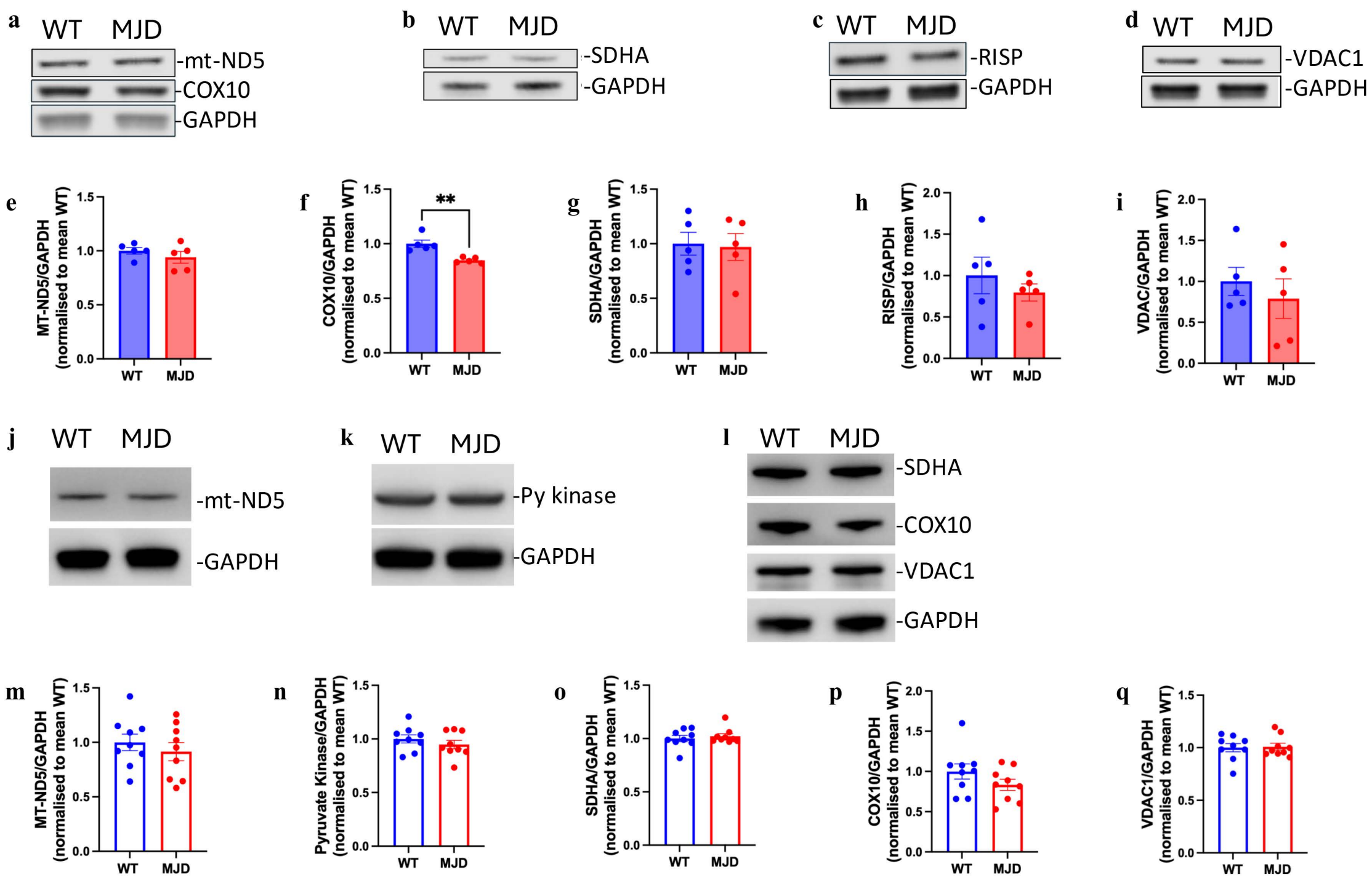

Fig. S2. Representative western blot membrane images from spinal cord samples extracted from 16-week-old CMVMJD135 mice showing (a) MT-ND5 and COX 10, (b) SDHA, (c) RISP and (d) VDAC1. GAPDH is shown as the loading control for each corresponding blot. (e–i) Densitometric quantification of e) MT-ND5, f) COX 10, g) SDHA, h) RISP, i) VDAC1, each normalised to GAPDH and shown relative to the mean WT group result. Data are from n = 5 biological replicates for each. Statistical comparisons involved t-tests, and asterisks indicate statistically significant differences (**p<0.01;).

Also shown are representative western blot membrane images from brain samples extracted from 20–25-week-old CMVMJD135 mice showing j) MT-ND5, k) Pyruvate kinase (Py kinase), l) SDHA, COX10 and VDAC1. GAPDH is shown as the loading control for each corresponding blot. (m–q) Densitometric quantification of m) MT-ND5, n) Pyruvate kinase (Py kinase), o) SDHA, p) COX10 and q) VDAC1, each normalised to GAPDH and shown relative to the mean WT group result. Data are from n = 9 biological replicates for each and statistical comparisons involved t-tests.

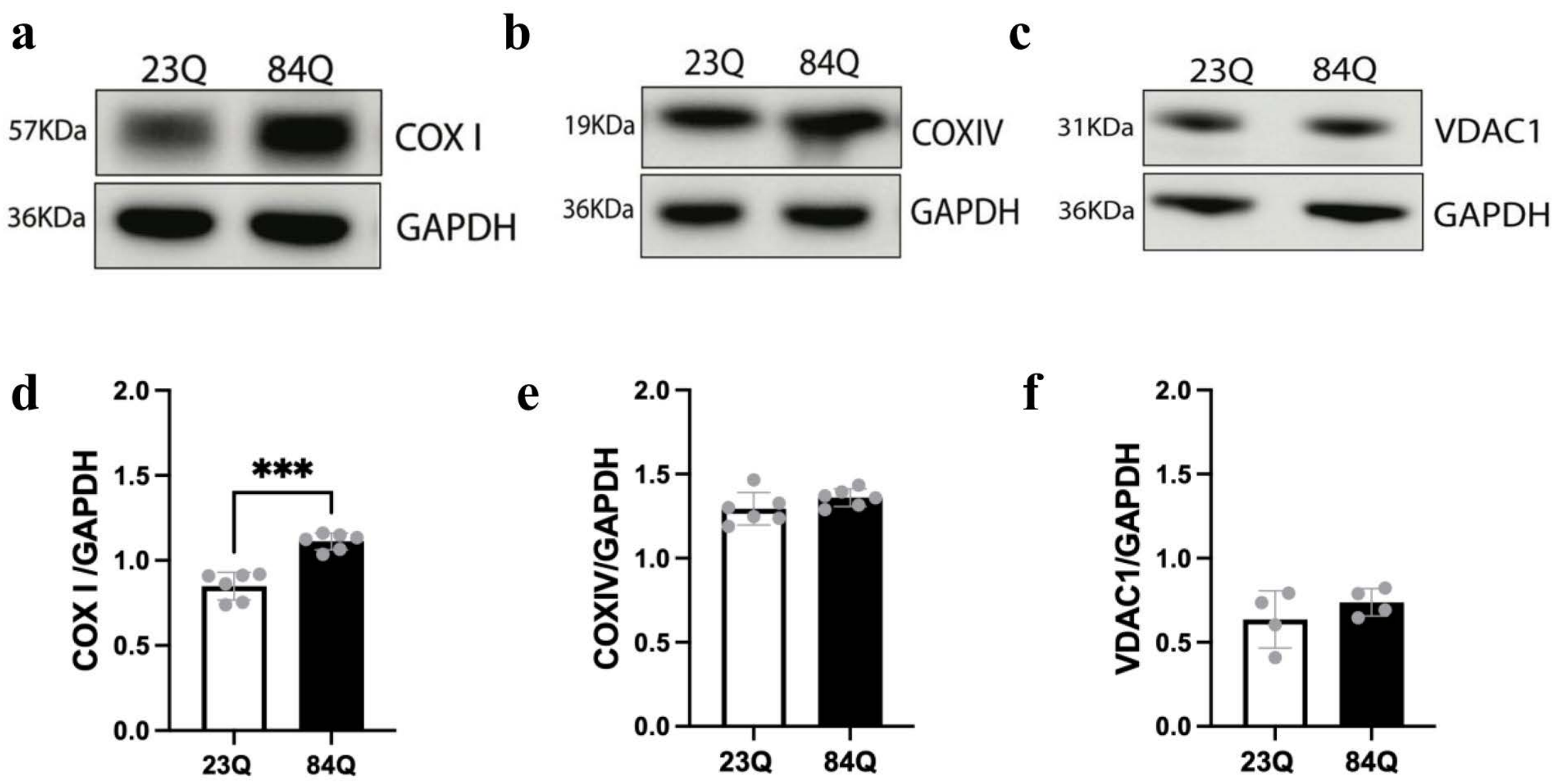

Fig. S3. Representative western blot membrane images from 5 dpf zebrafish expressing human hATXN3 23Q (WT) or 84Q (mutant) showing COX I (a), COX IV (b), and VDAC1 (c). GAPDH is shown as the loading control for each corresponding blot. (d–f) Densitometric quantification of COX I (d), COX IV (e), and VDAC1 (f), normalised to GAPDH. Each data point represents one biological replicate consisting of a pooled sample of 20 zebrafish embryos. Data are from n = 6 biological replicates for COX I and COX IV, and n = 4 for VDAC1. Statistical comparisons involved t-tests, *** indicates statistically significant difference ($p < 0.001$).

Disease Models & Mechanisms • Supplementary information

For Figure 2

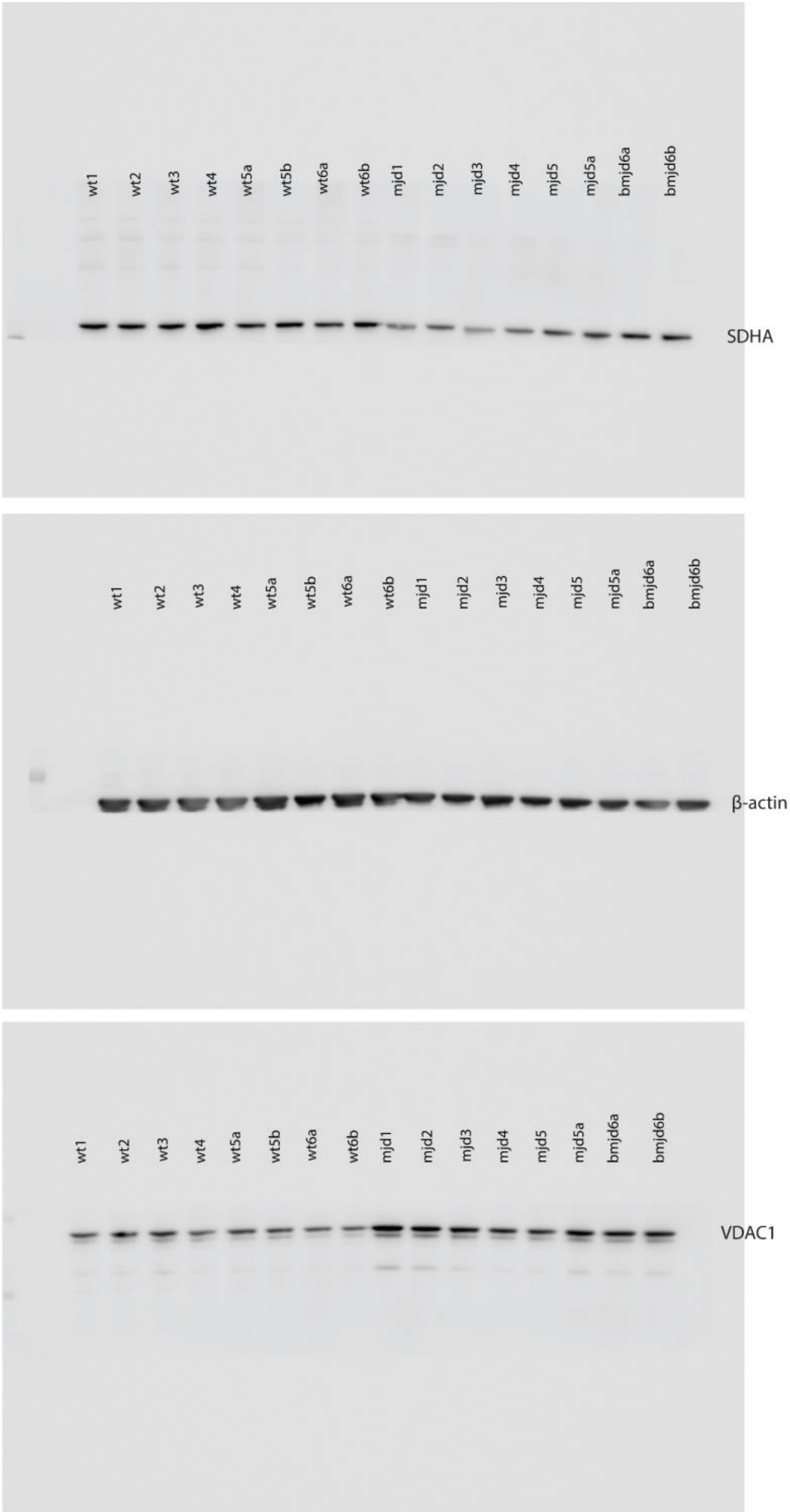
wt1 wt2 wt3 wt4 wt5a wt5b wt6a wt6b mjd1 mjd2 mjd3 mjd4 mjd5 mjd5a bmjd6a bmjd6b
SDHA
wt1 wt2 wt3 wt4 wt5a wt5b wt6a wt6b mjd1 mjd2 mjd3 mjd4 mjd5 mjd5a bmjd6a bmjd6b
β-actin
wt1 wt2 wt3 wt4 wt5a wt5b wt6a wt6b mjd1 mjd2 mjd3 mjd4 mjd5 mjd5a bmjd6a bmjd6b
VDAC1

ɔr Figure 2

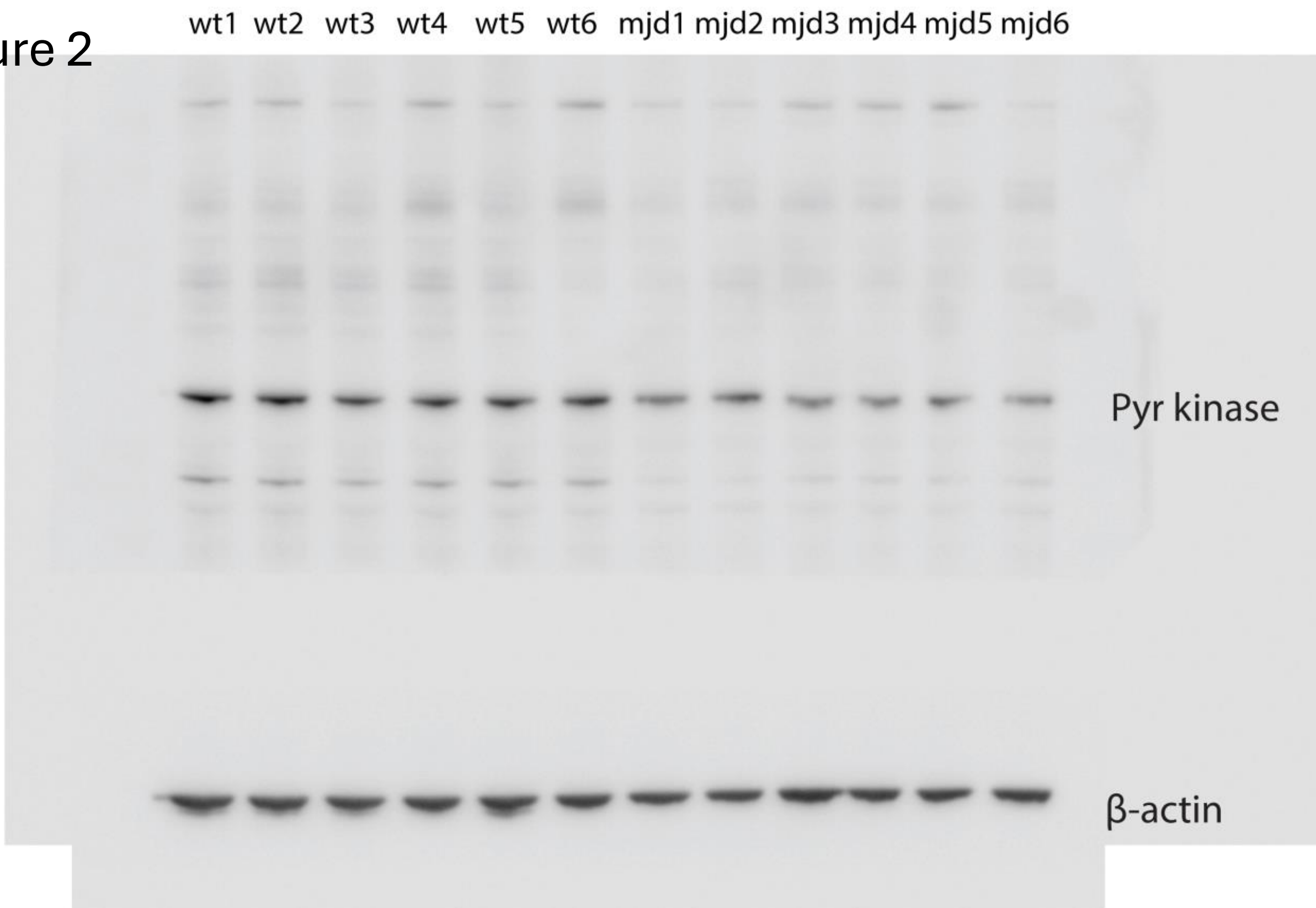

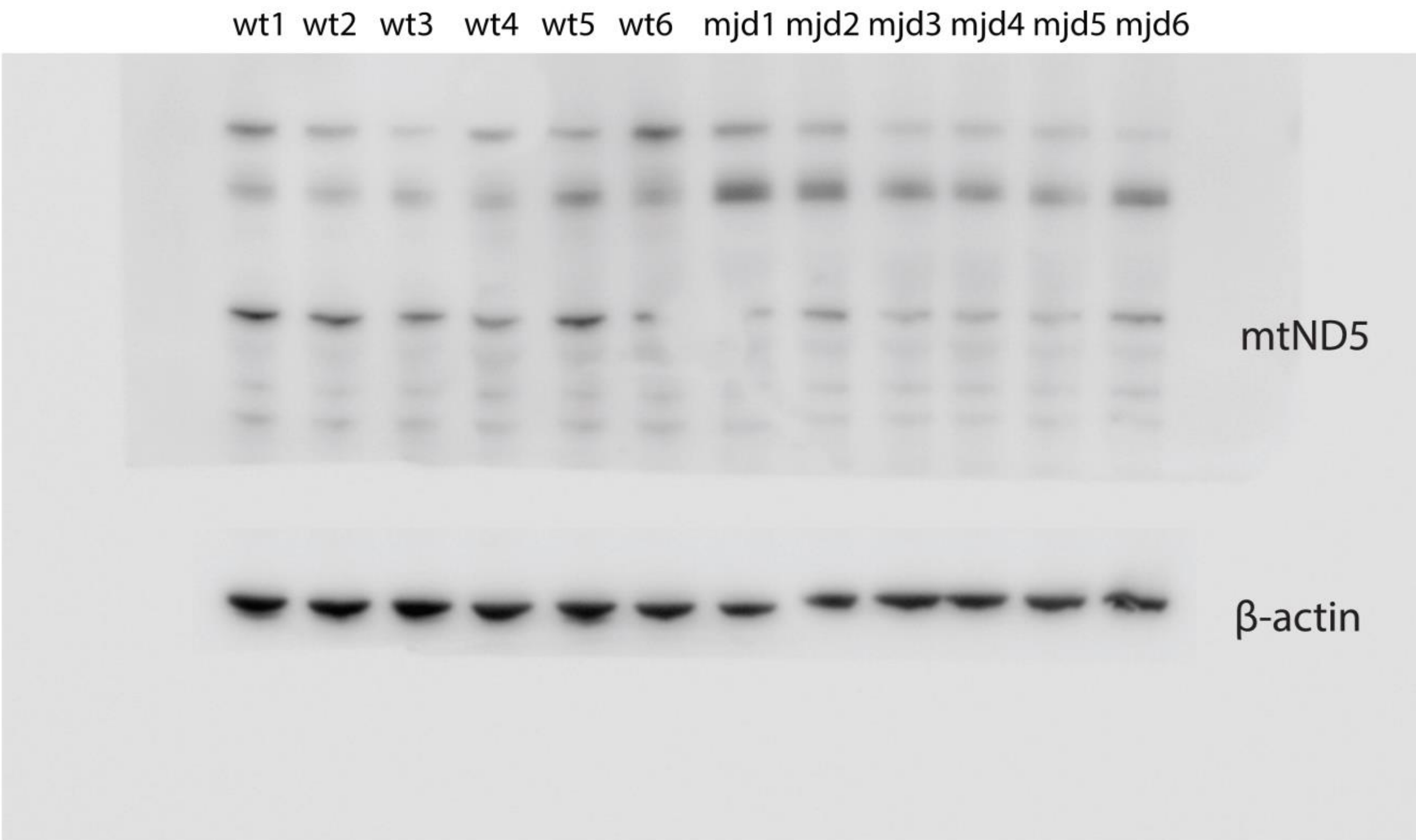

Disease Models & Mechanisms • Supplementary information

For Figure 2

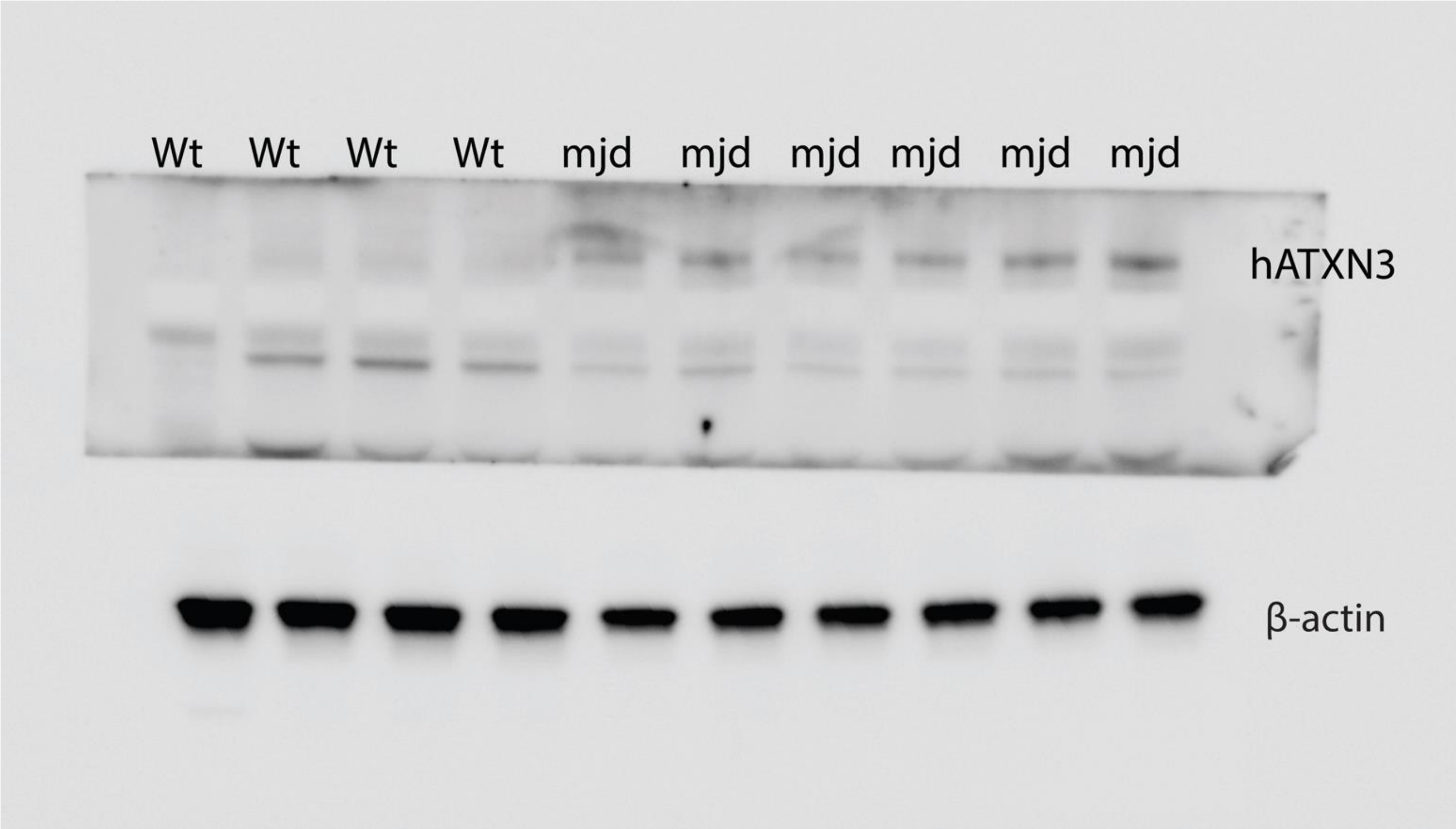

For Figure 4

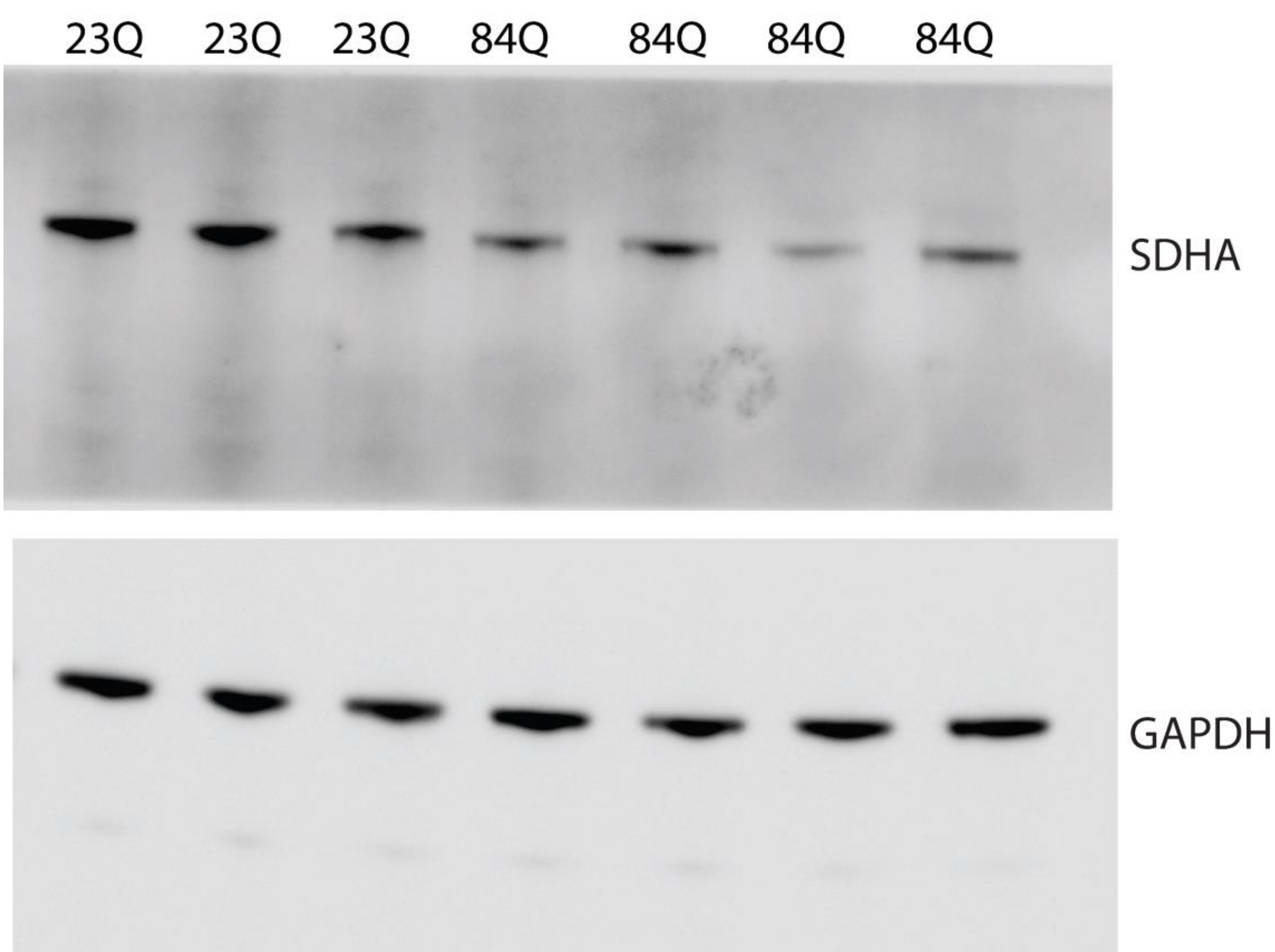

For Supplementary Figure 2

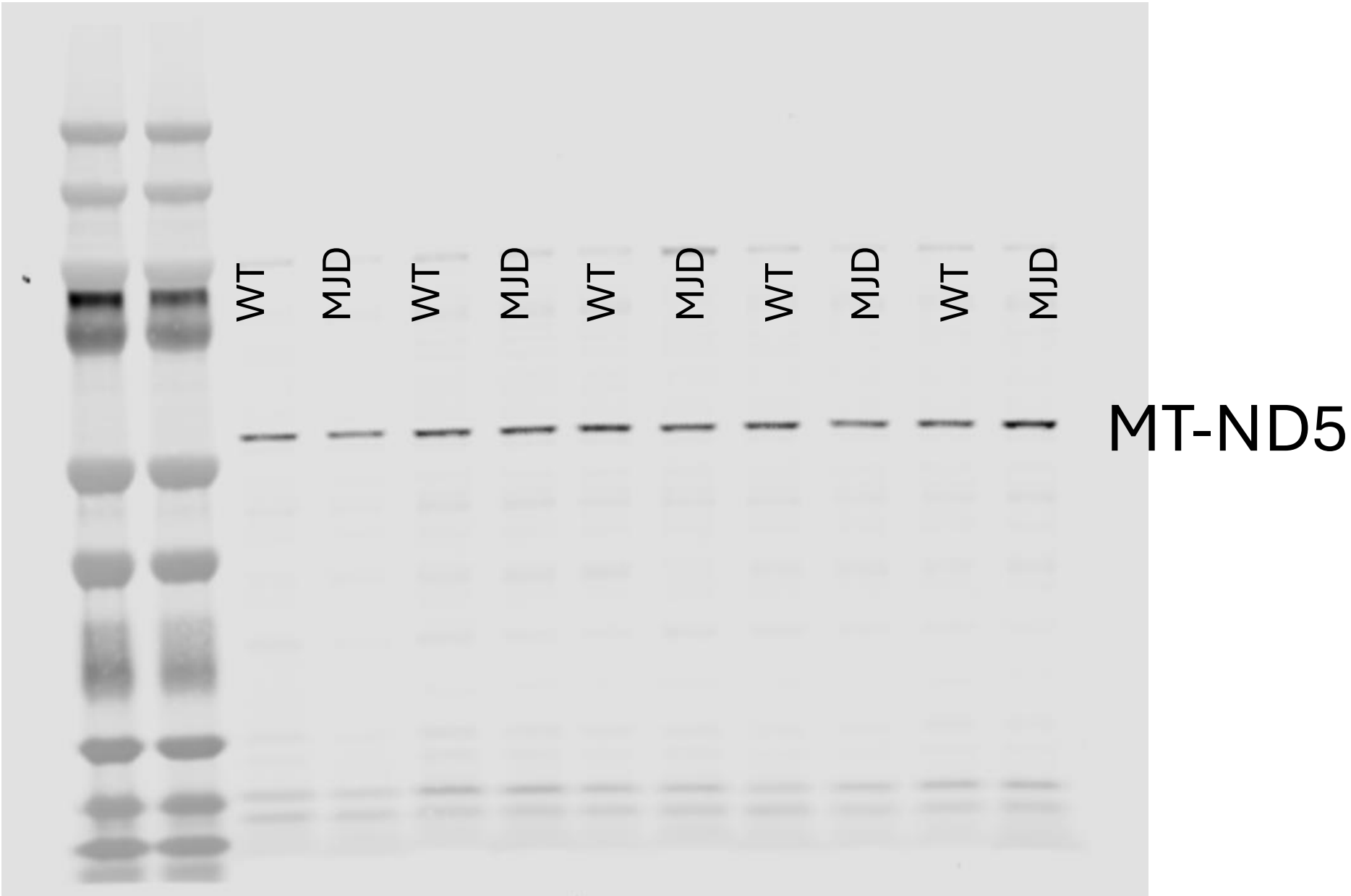

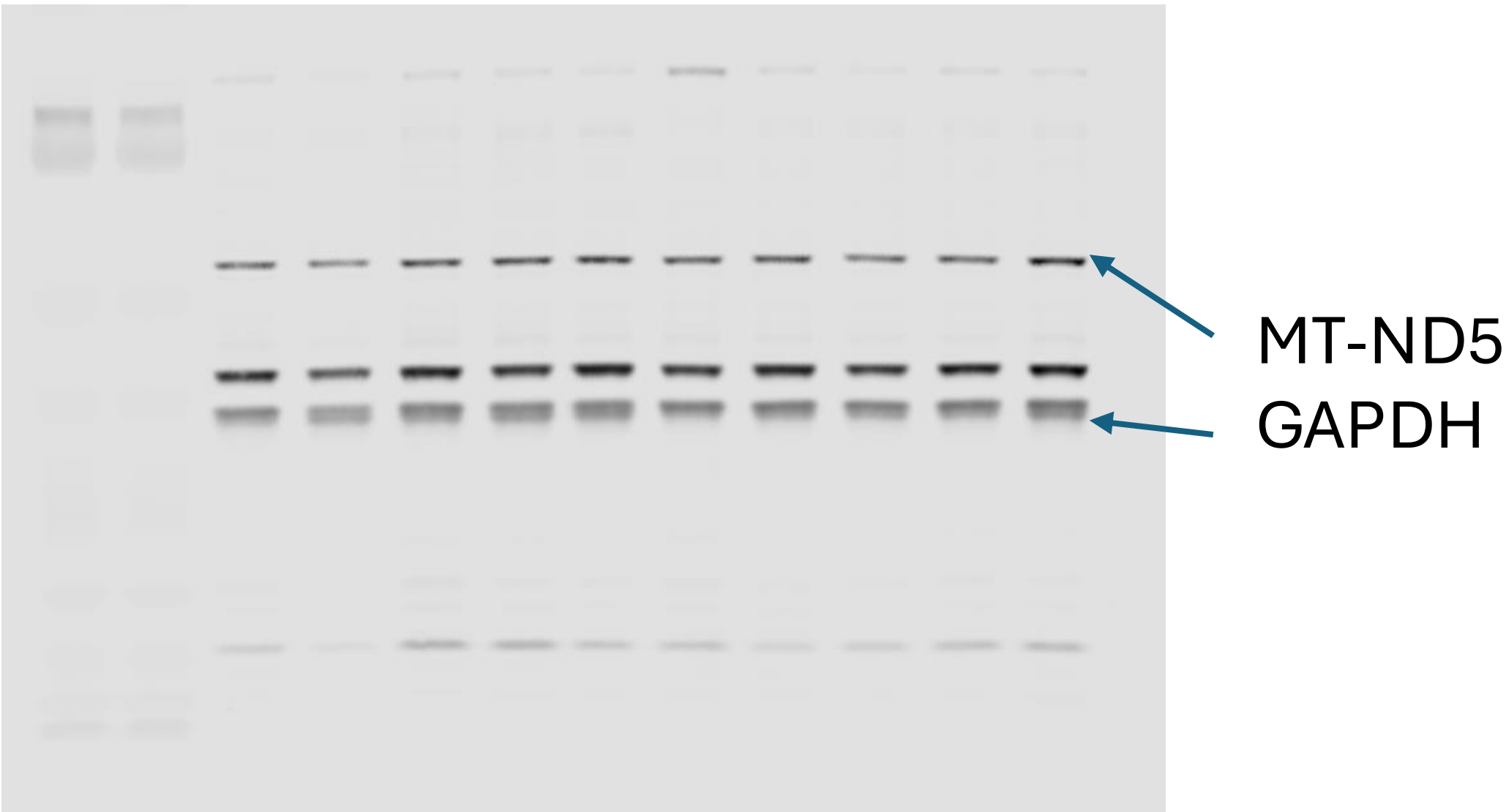

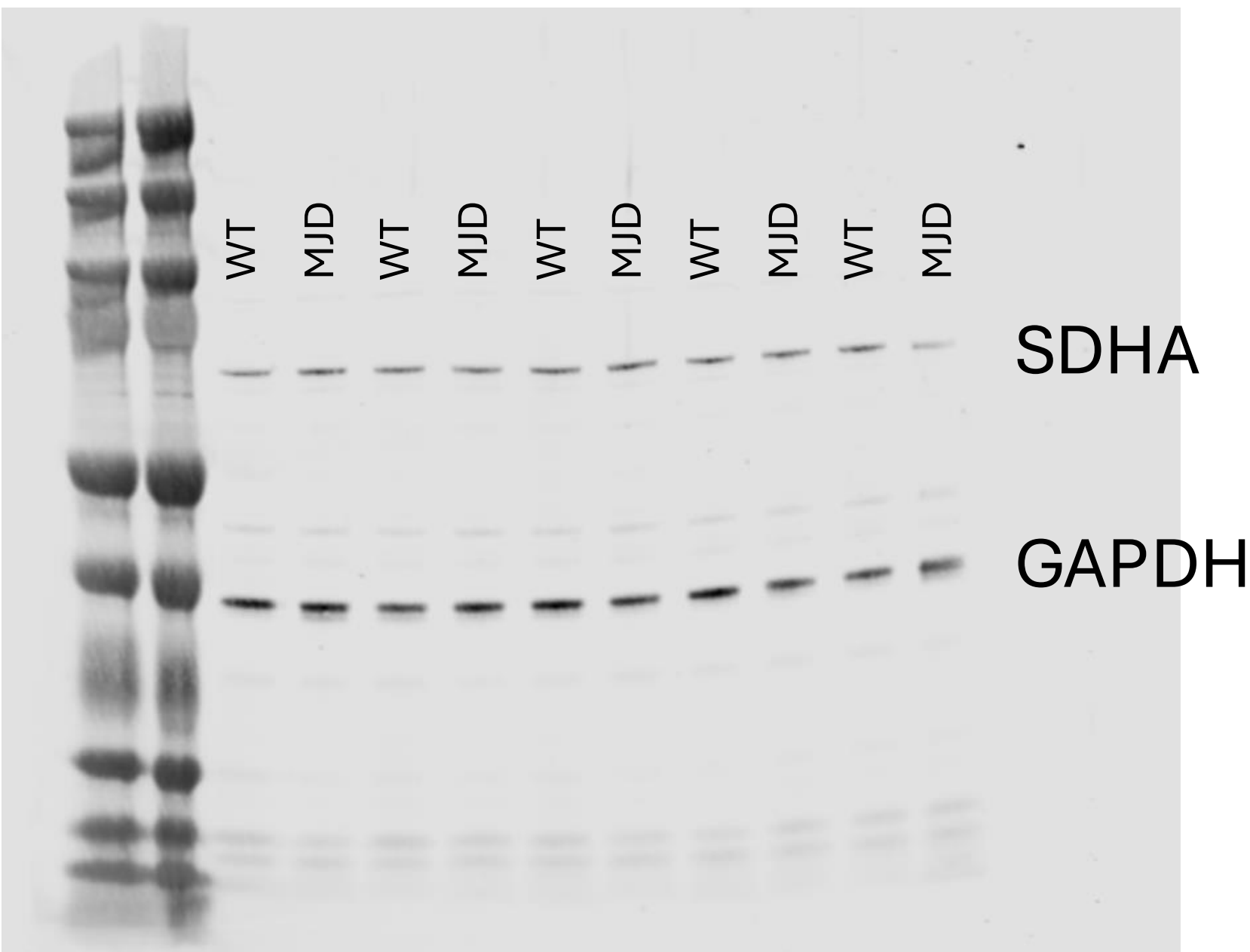

Disease Models & Mechanisms • Supplementary information

For Supplementary Figure 2

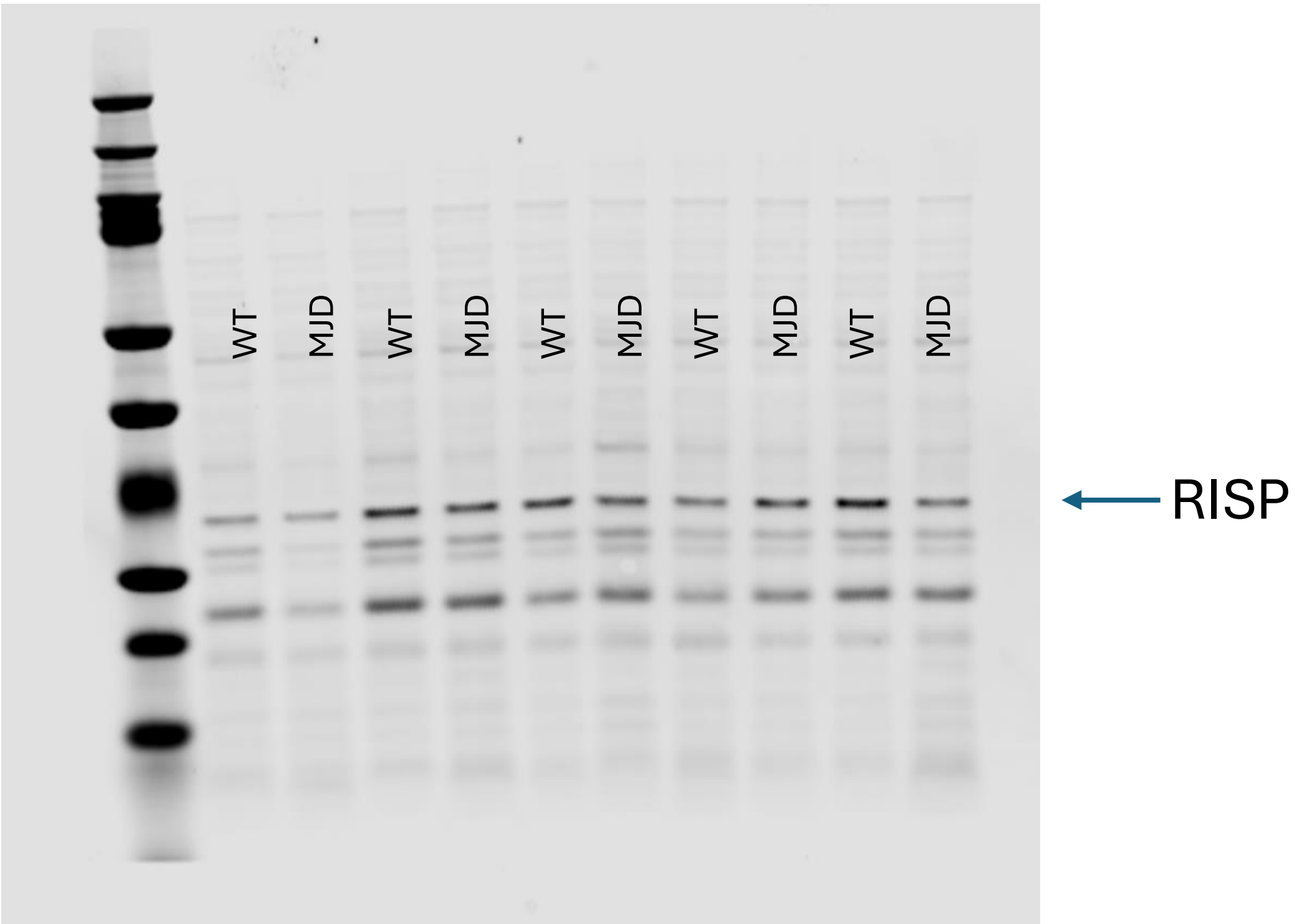

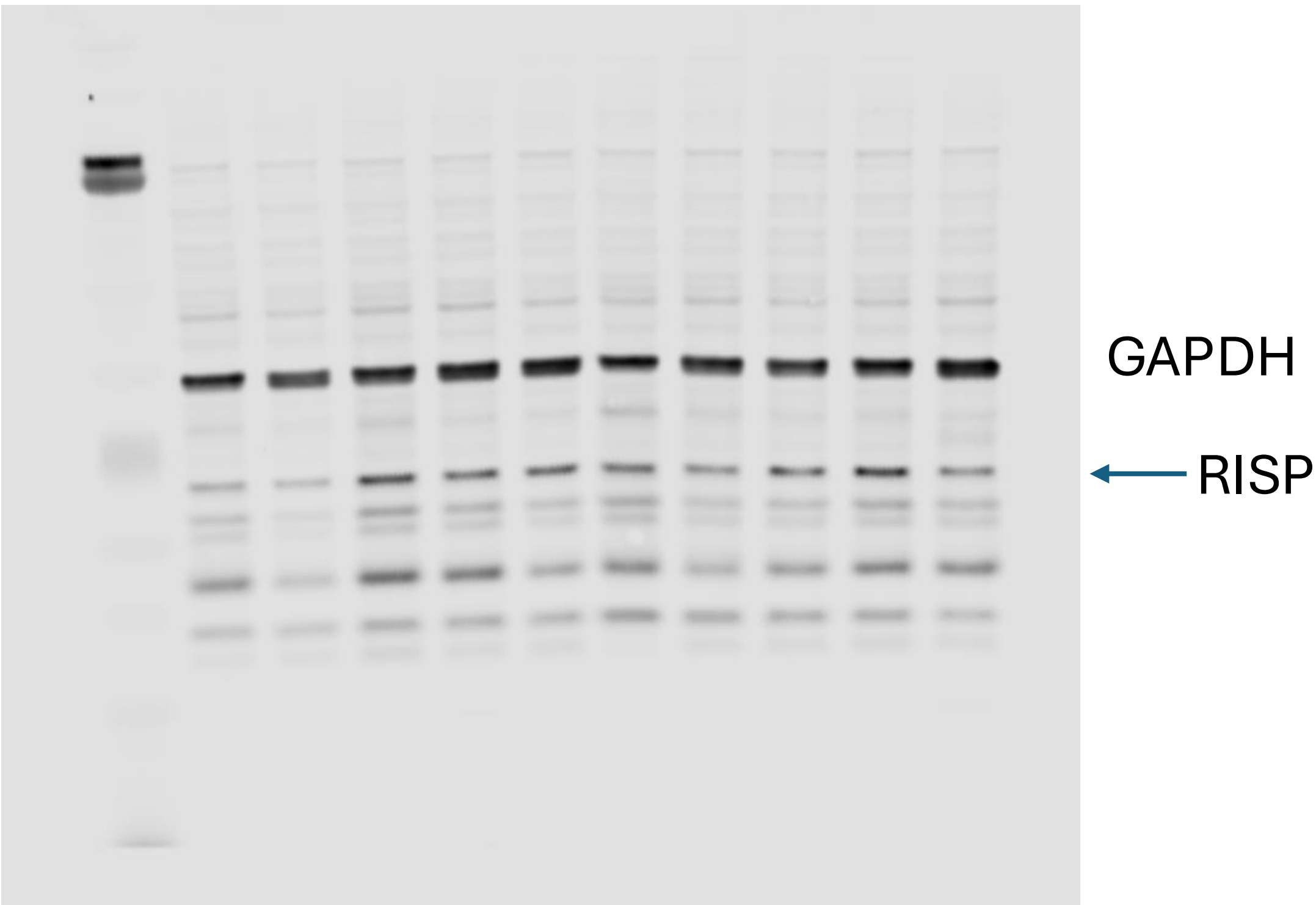

For Supplementary Figure 2

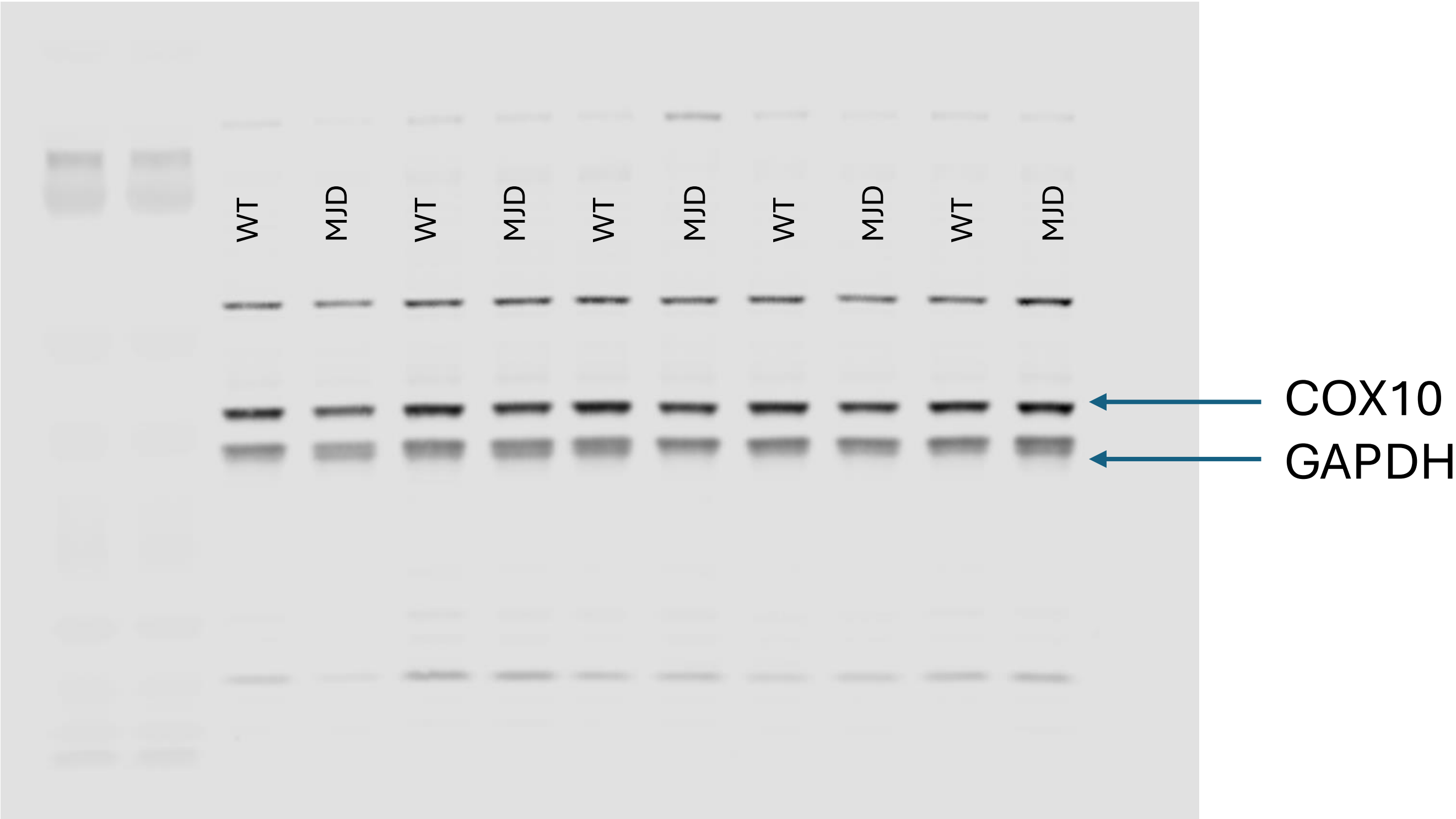

For Supplementary Figure 2

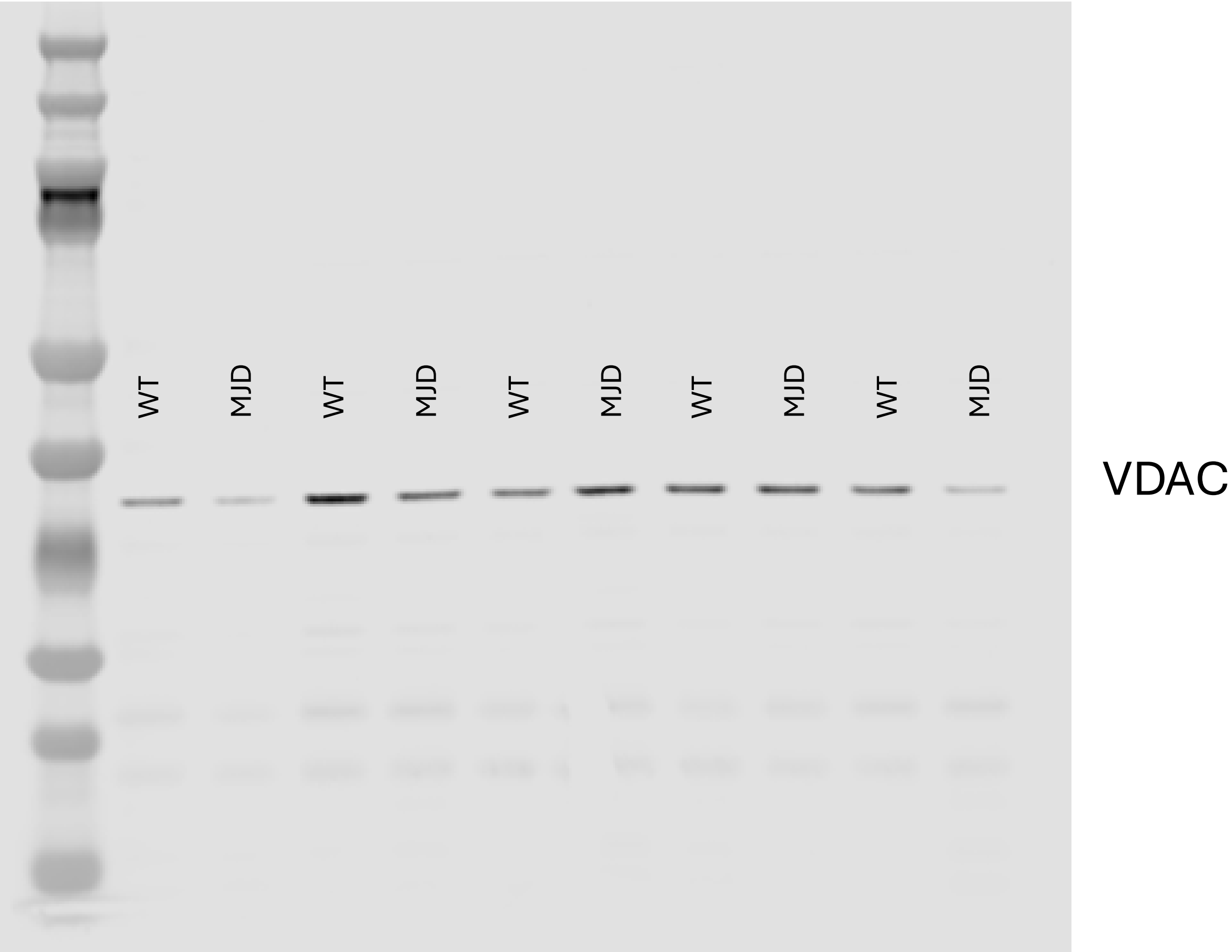

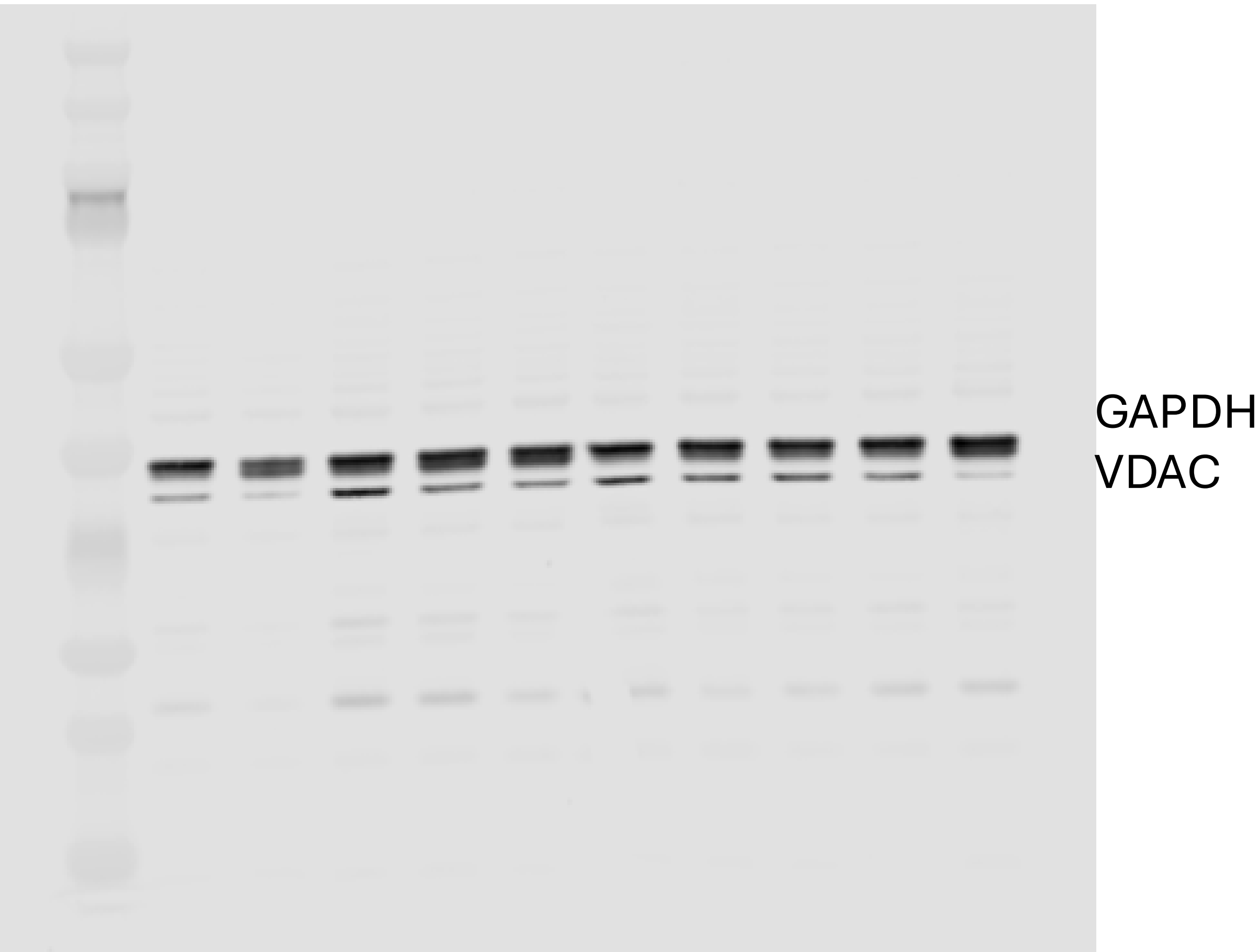

Disease Models & Mechanisms • Supplementary information

For Supplementary Figure 2

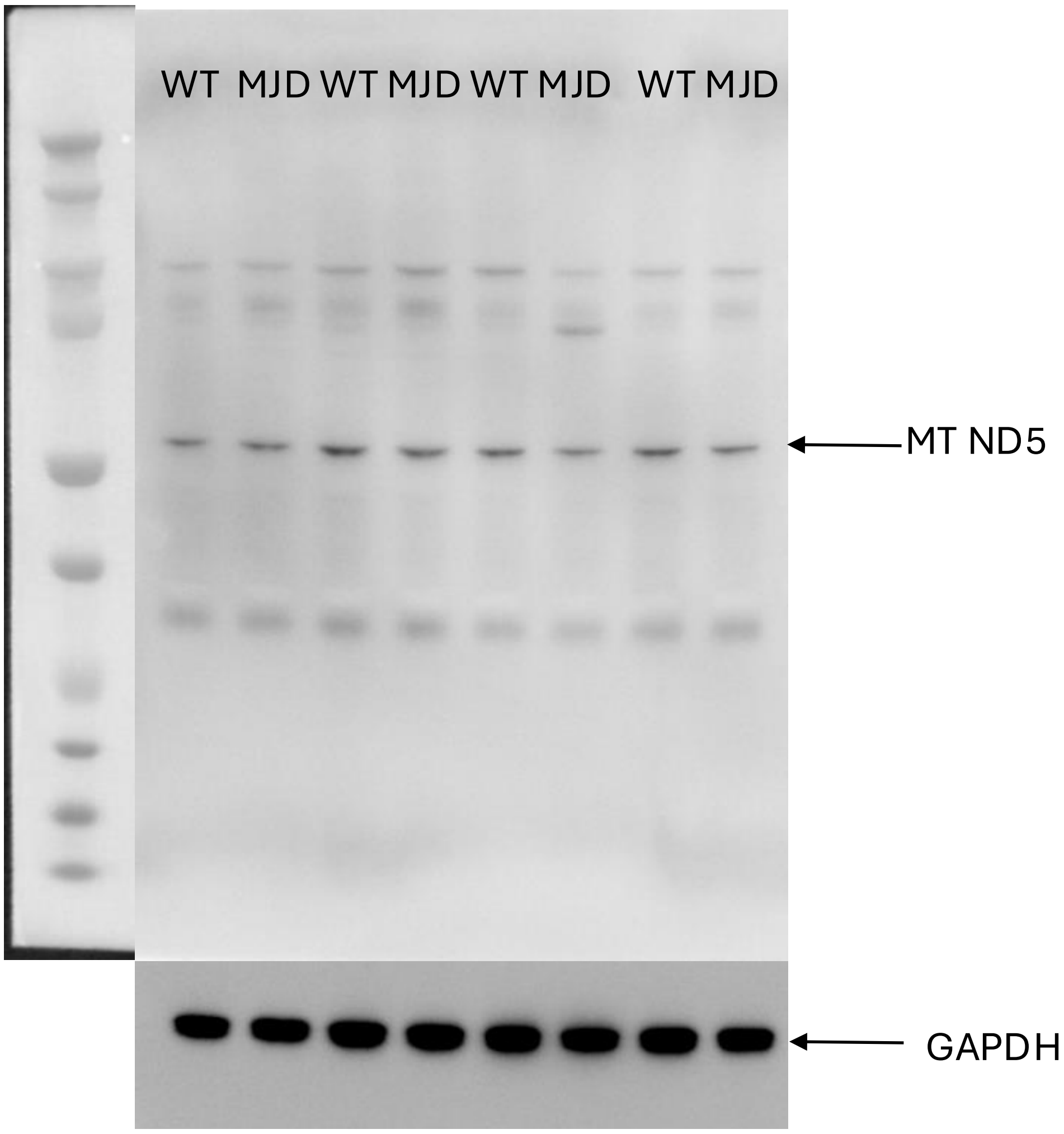

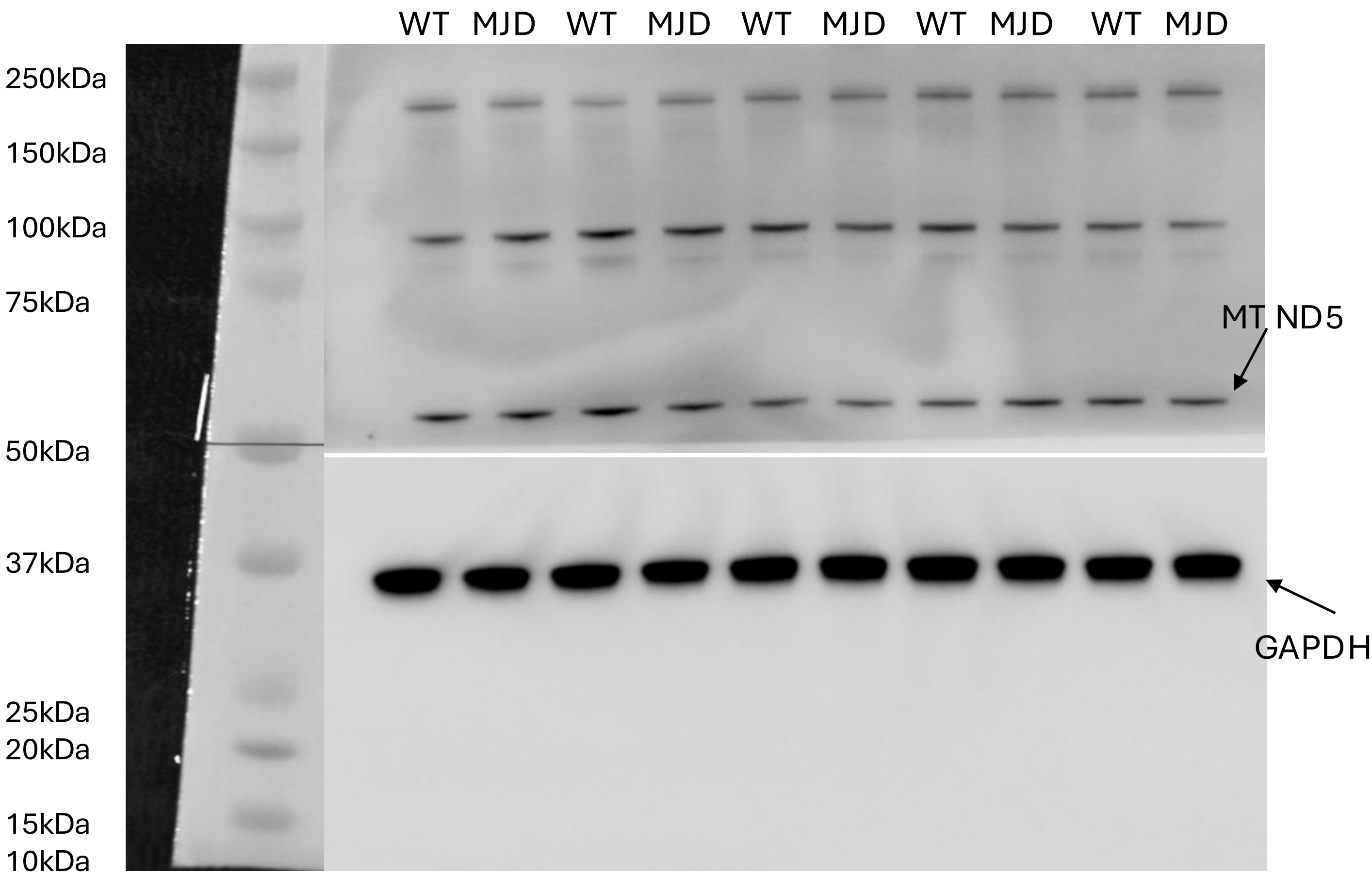

For Supplementary Figure 2

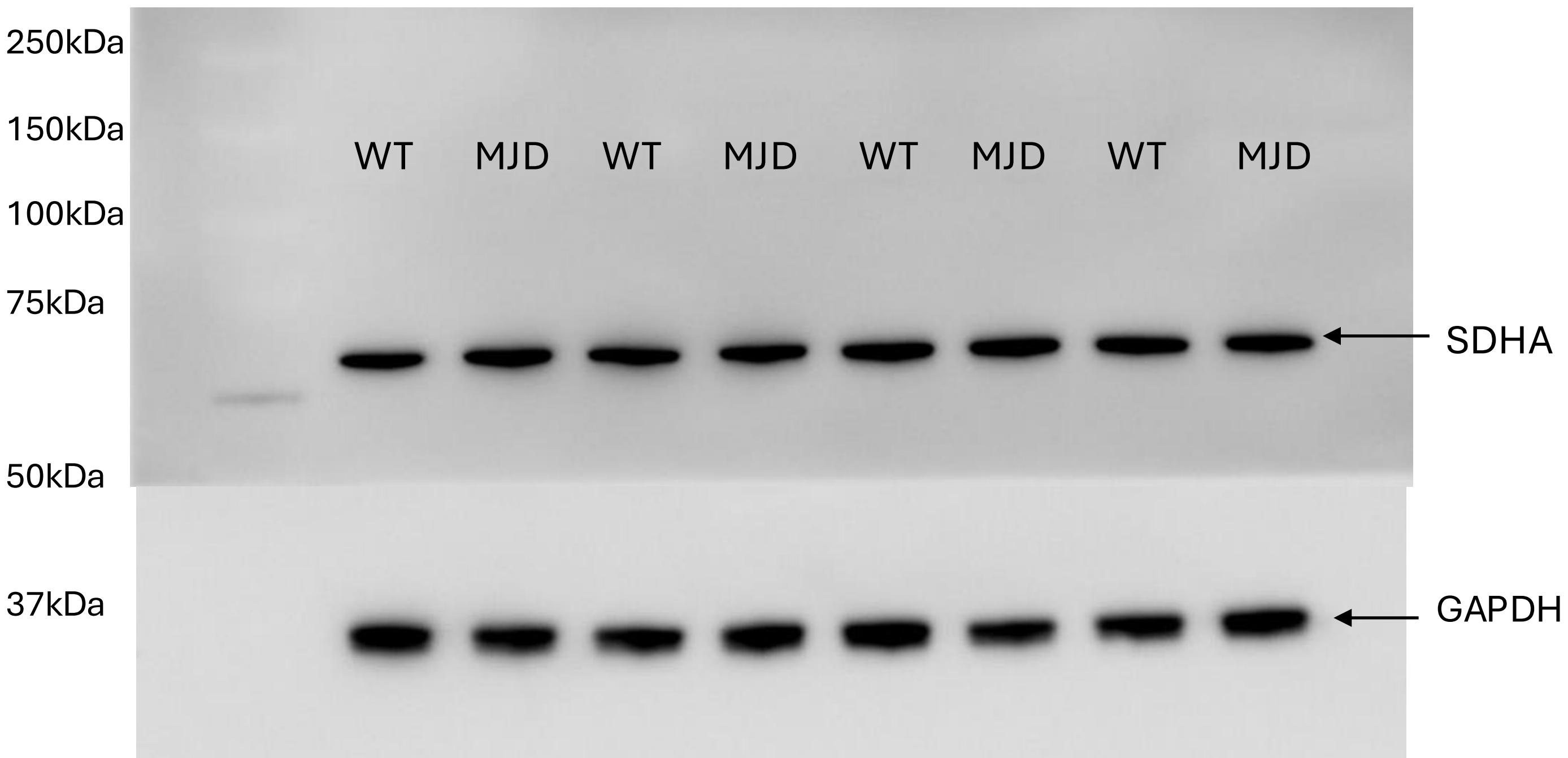

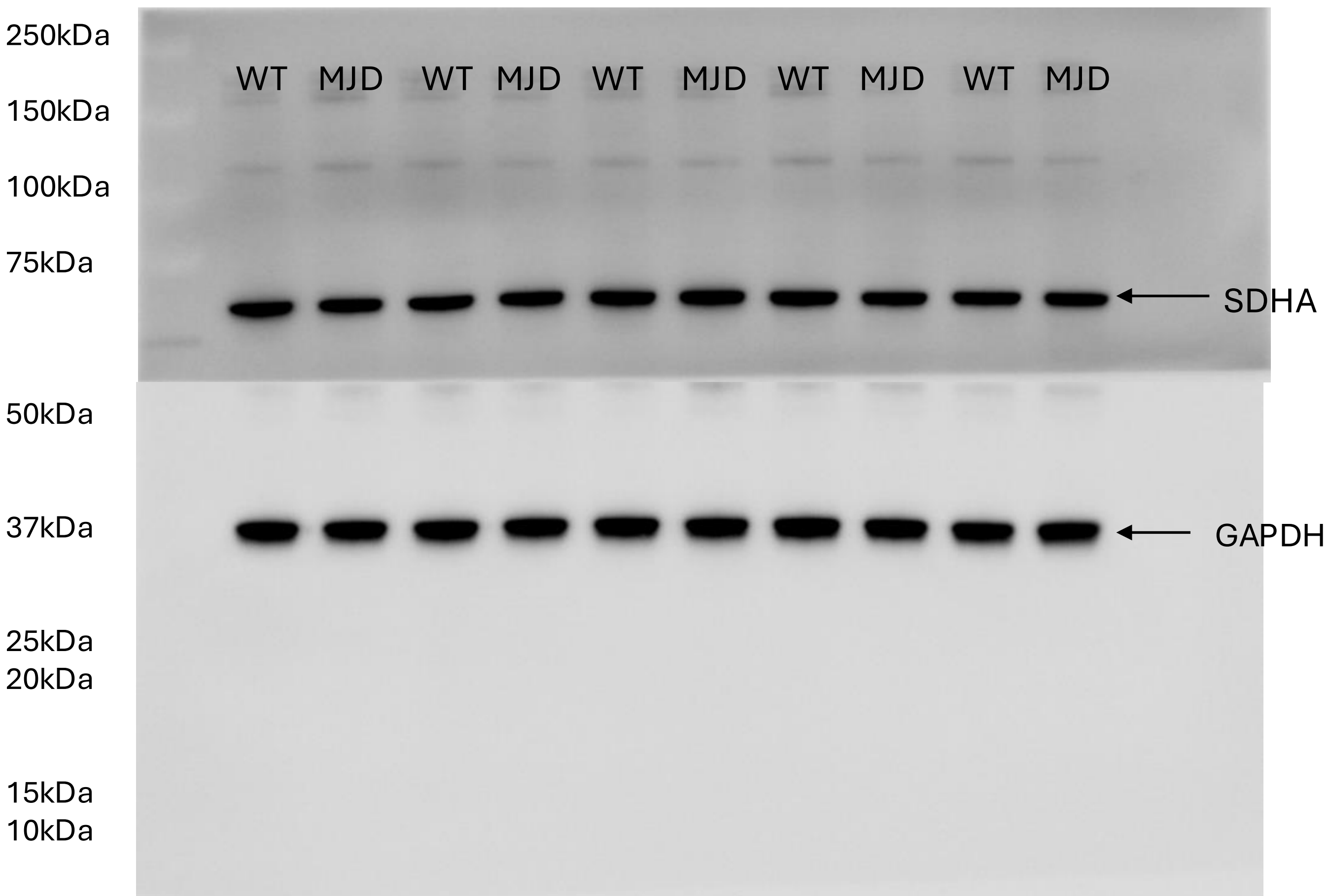

For Supplementary Figure 2

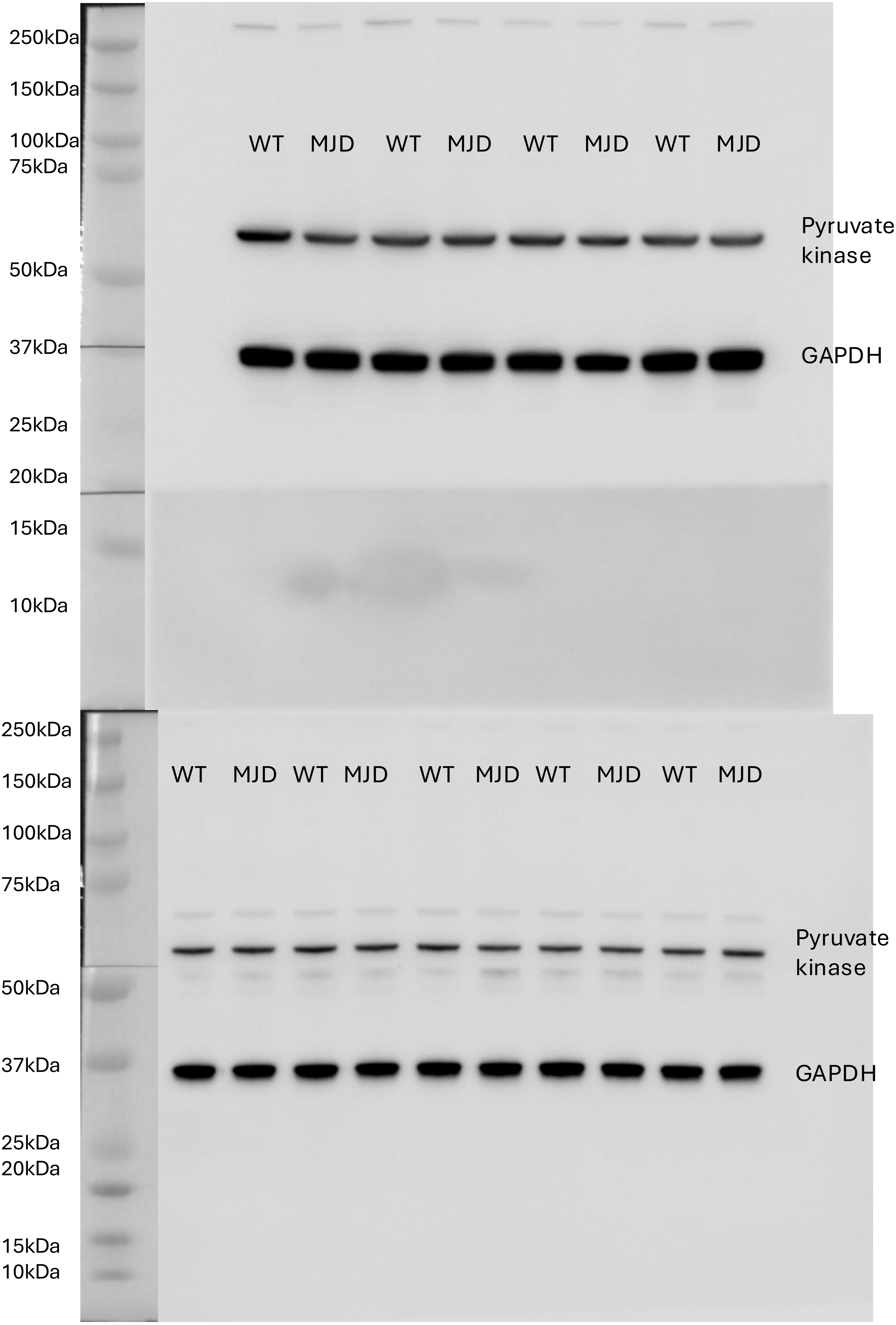

For Supplementary Figure 2

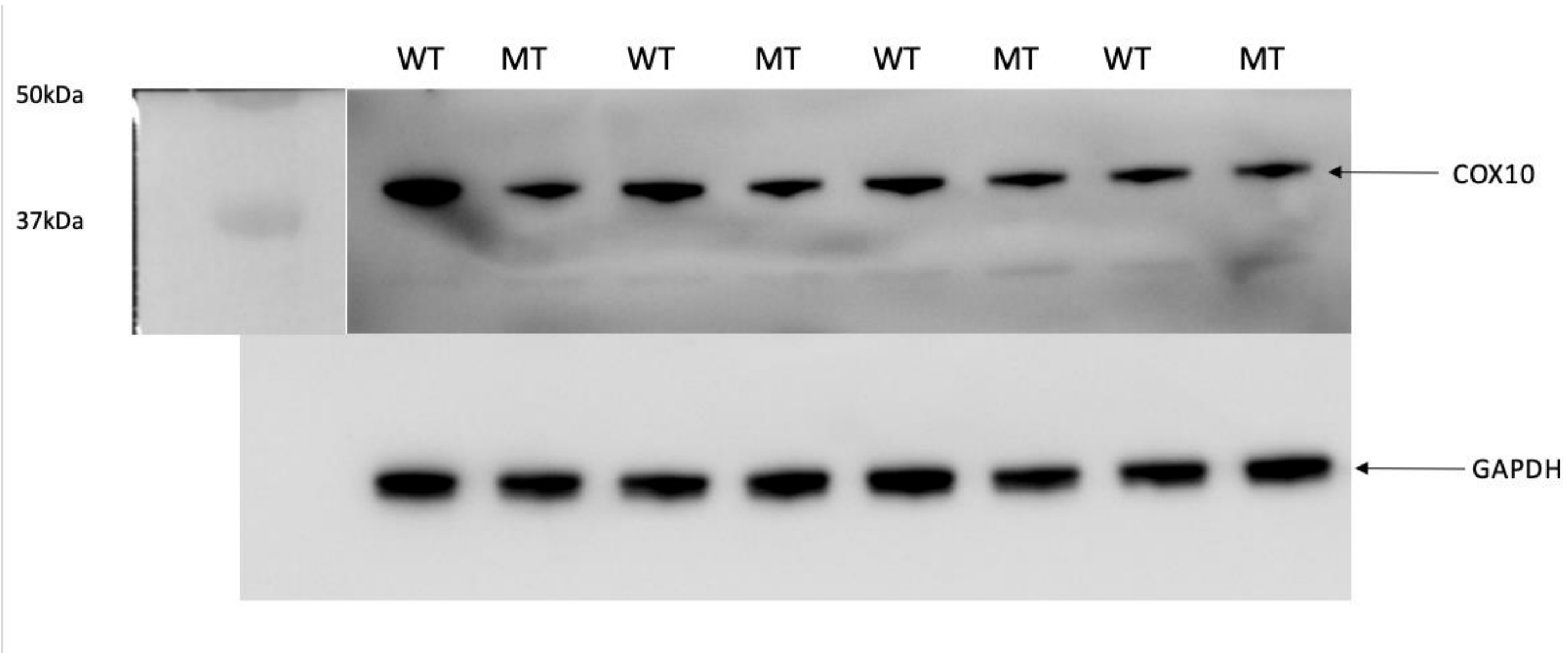

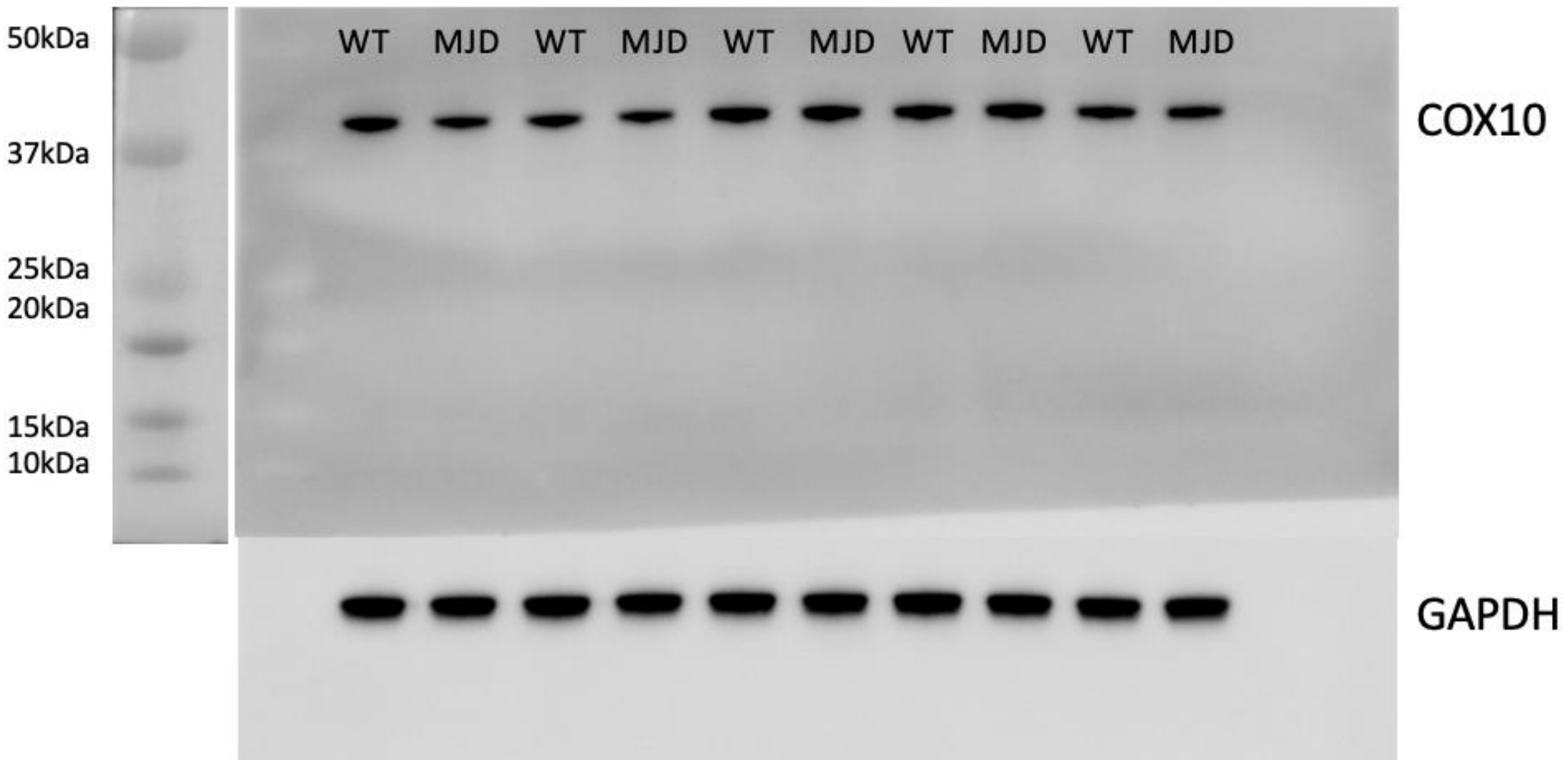

Disease Models & Mechanisms • Supplementary information

For Supplementary Figure 2

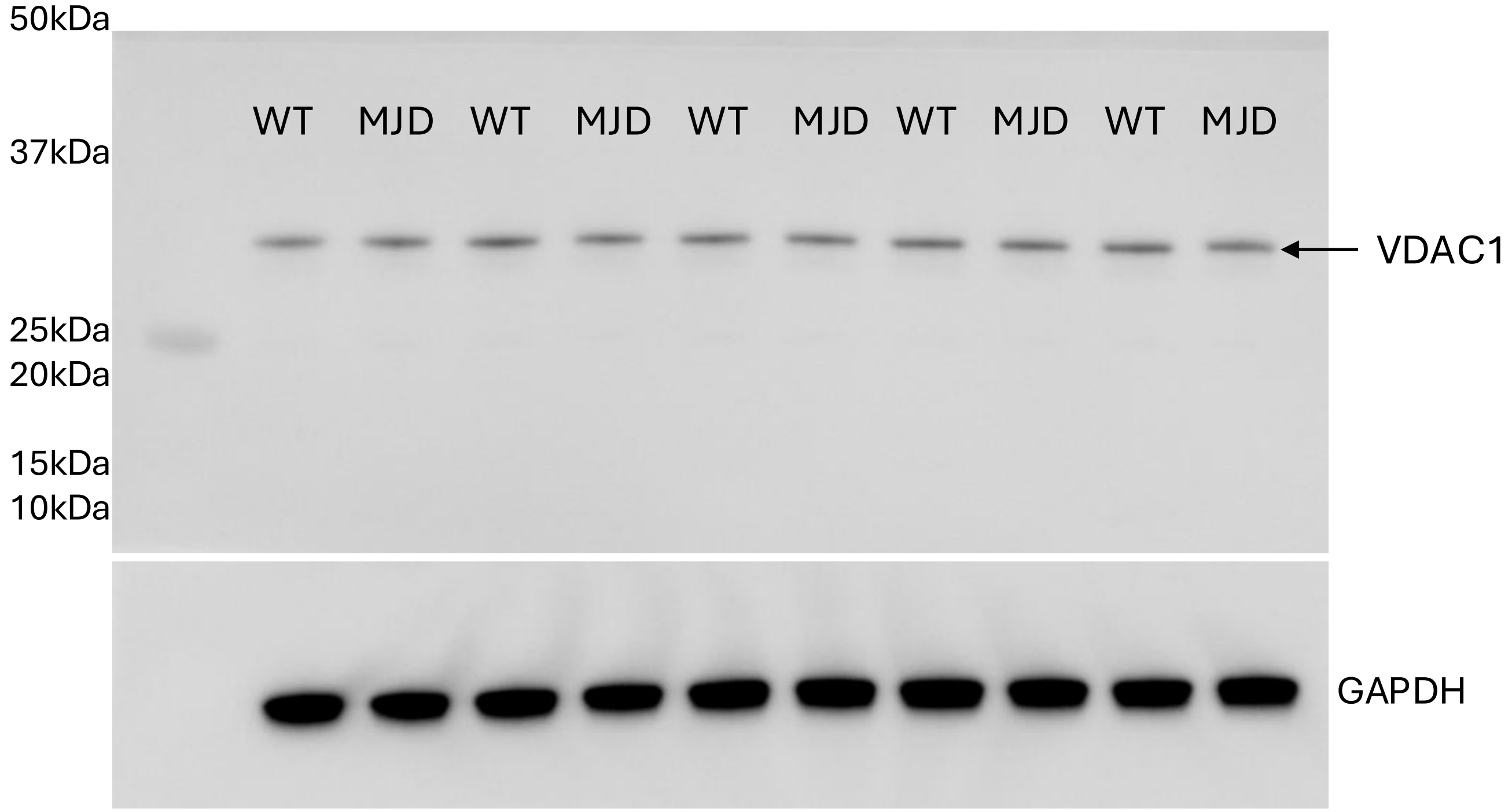

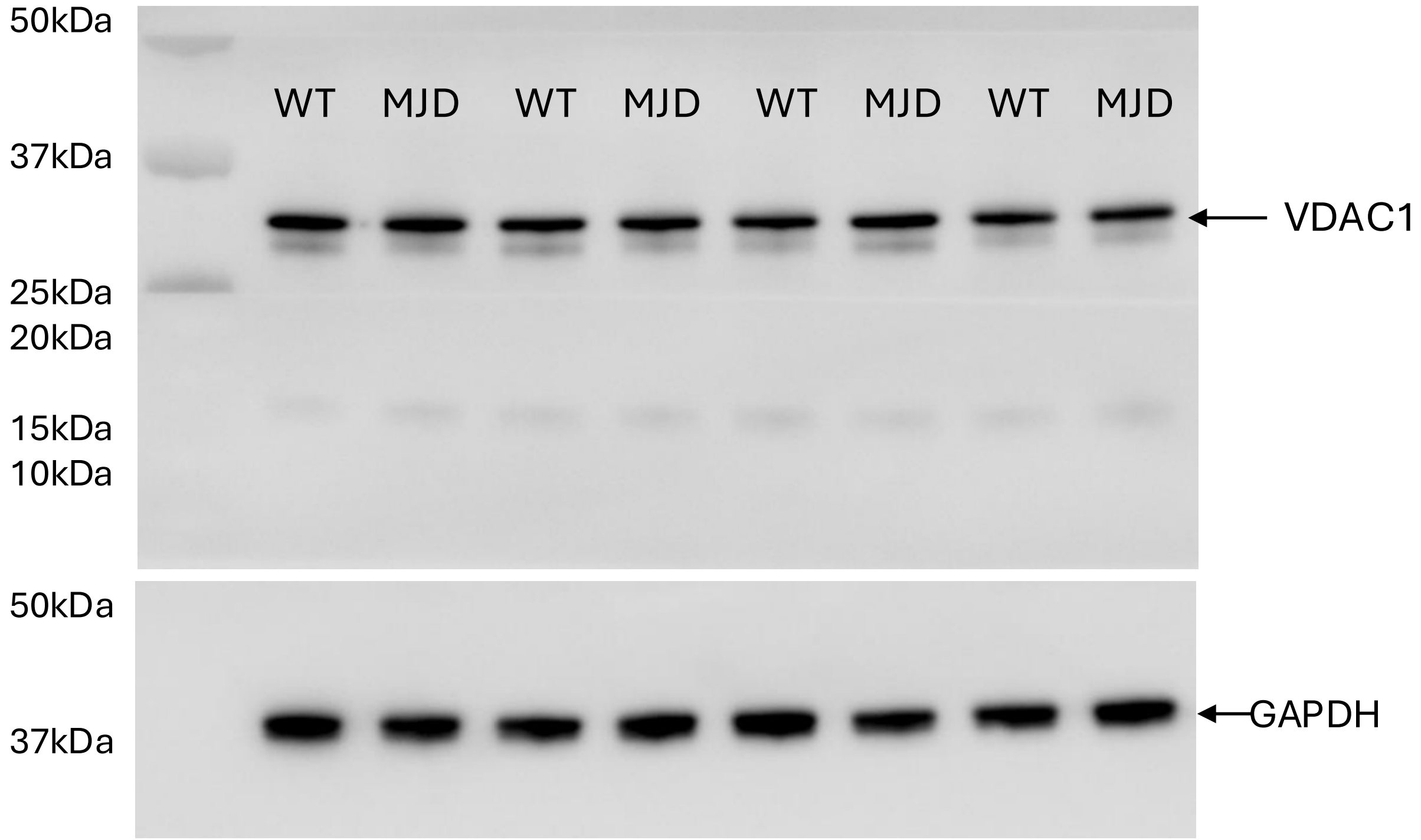

Disease Models & Mechanisms • Supplementary information

For Supplementary Figure 3- Zebrafish western blots

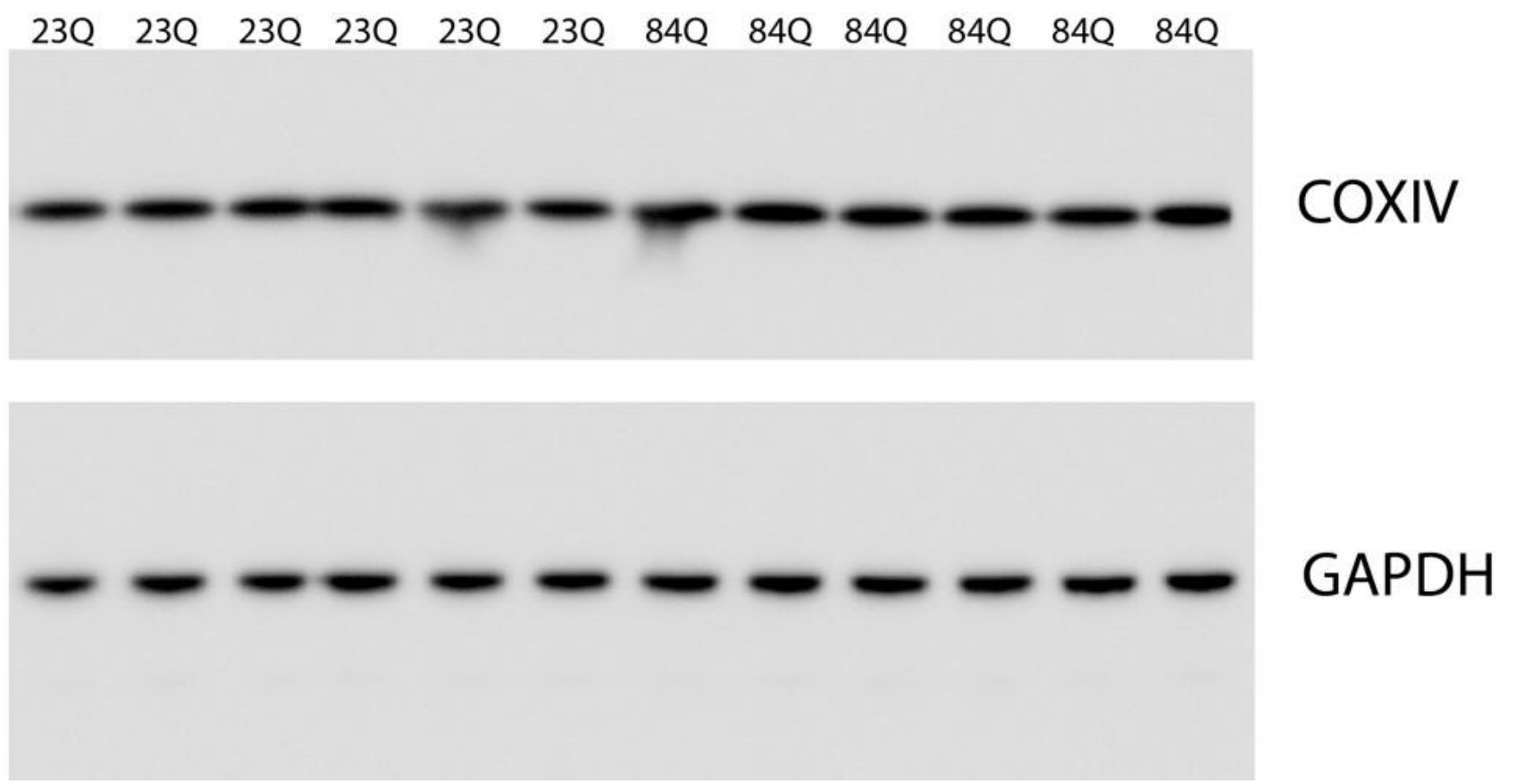

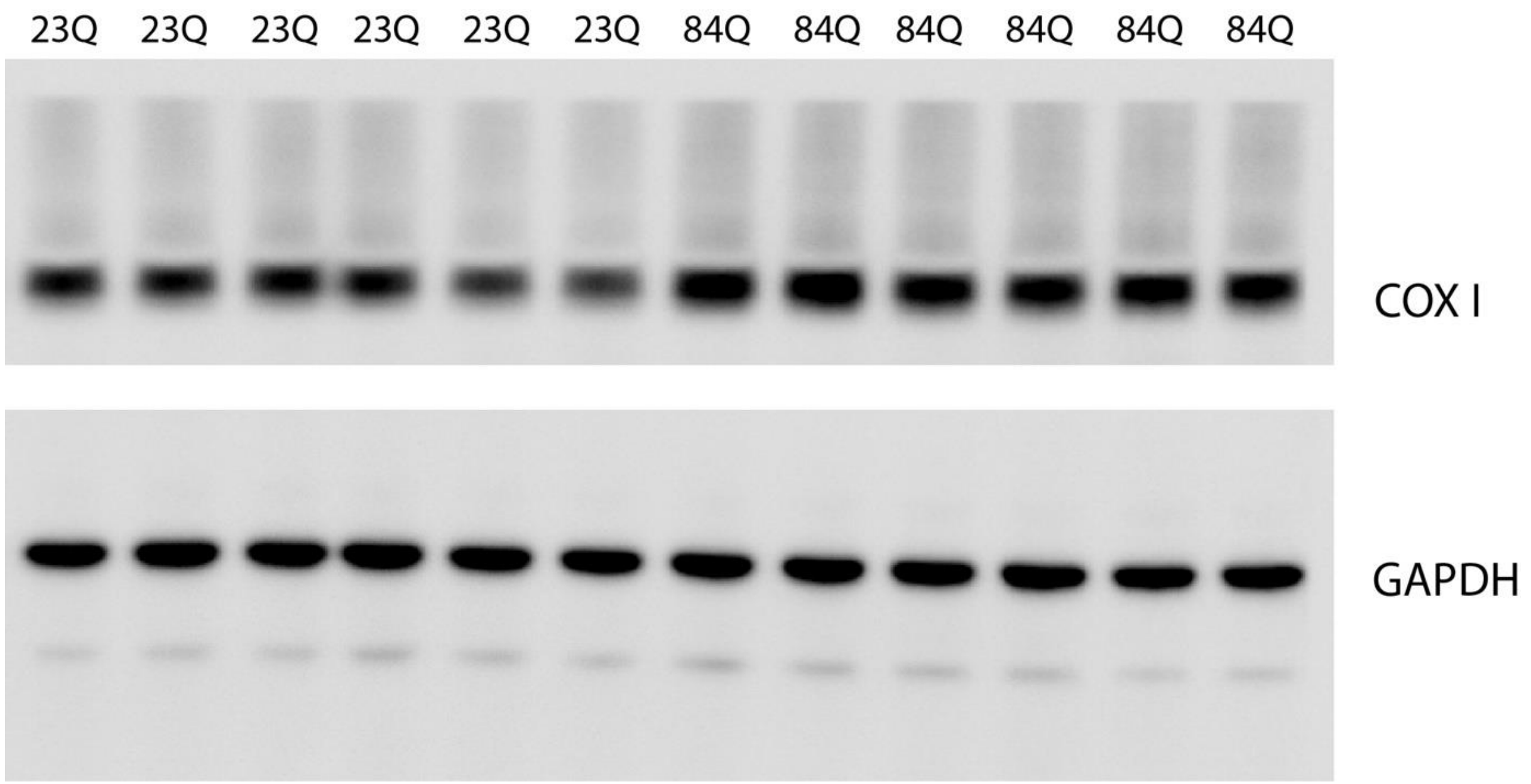

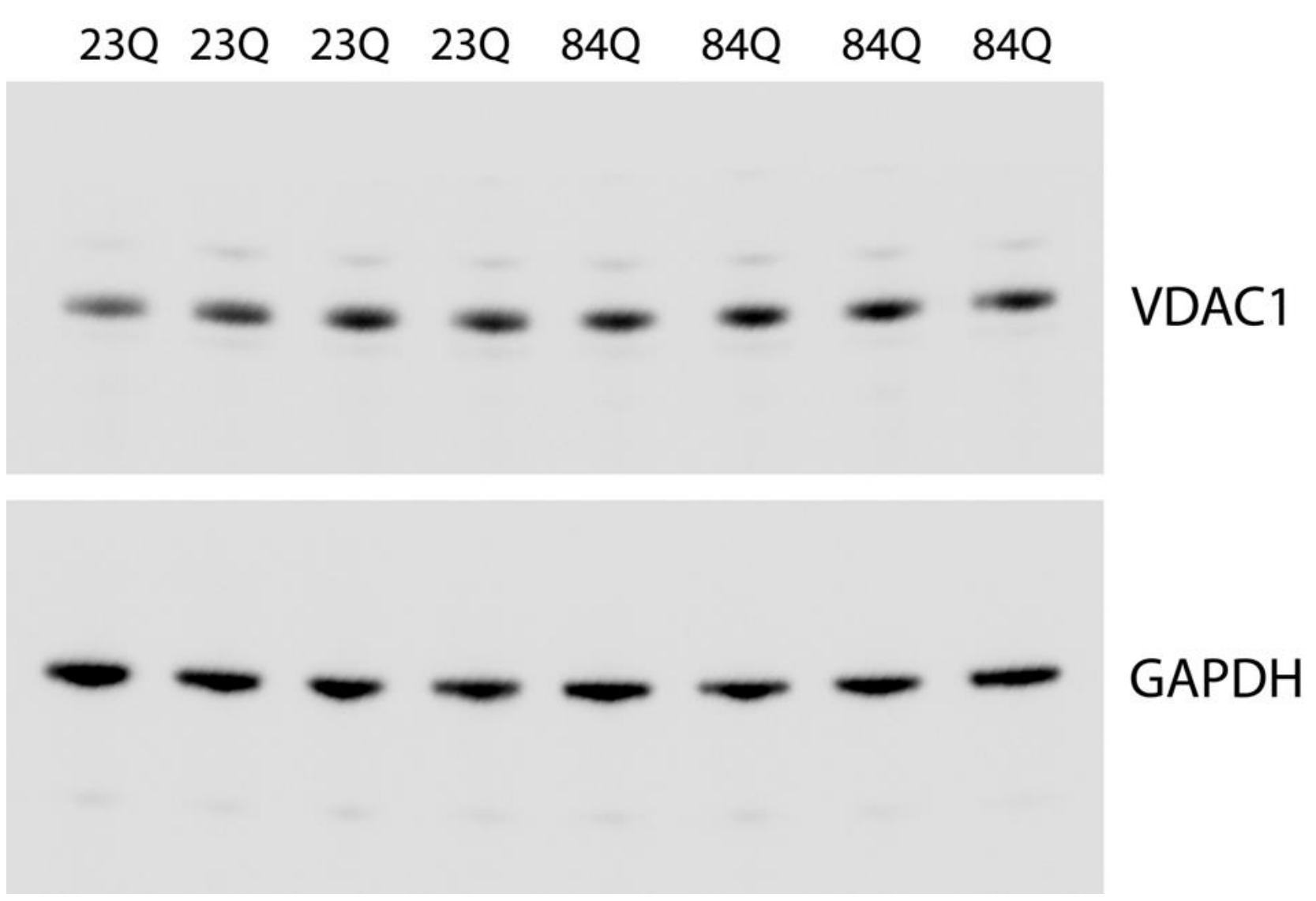

Fig. S4. Blot transparency.

Table S1. Oligonucleotide primer sequences used for qPCR analysis.

Primer	Forward (5' - 3')	Reverse (5' - 3')
ms*gapdh*	TGCGACTTCAACAGCAACTC	GCCTCTCTTGCTCAGTGTCC
zf*gapdh*	CGCTGGCATCTCCCTCAA	TCAGCAACACGATGGCTGTAG
mtDNA	CCAGATGCACCTGAGCTAATAA	CGTATCGGAATCGTGGGTATG
ndufs4	TGTAGGCTGGCAGAGGGAC	GACAGGCCGAAACAGGATG
mtnd1	AGCCTACGCCGTACCAGTATT	GTTTCACGCCATCAGCTACTG
cox5ab	GTCACCGGAGCTTCAGGAT	CGAGCCGAGAGGTAGAAAAAC
atp5α1	TTCTTGGAGCCGACACTGG	CGAACACCACAACACCAACG
